# The school bus routing and scheduling problem with transfers

**DOI:** 10.1002/net.21589

**Published:** 2015-02-02

**Authors:** Michael Bögl, Karl F. Doerner, Sophie N. Parragh

**Affiliations:** ^1^ Christian Doppler Laboratory for efficient intermodal transport operationsJohannes Kepler University LinzAltenberger Straße 69, 4040Linz, Austria; ^2^ Institute for Production and Logistics ManagementJohannes Kepler University LinzAltenberger Straße 69, 4040Linz, Austria; ^3^ Department of Business AdministrationUniversity of ViennaOskar‐Morgenstern‐Platz 1, 1090Vienna, Austria; ^4^ Institute for Transport and Logistics ManagementVienna University of Economics and BusinessWelthandelsplatz 1, 1020Vienna, Austria

**Keywords:** school bus routing, transfers, routing and scheduling, metaheuristic

## Abstract

In this article, we study the school bus routing and scheduling problem with transfers arising in the field of nonperiodic public transportation systems. It deals with the transportation of pupils from home to their school in the morning taking the possibility that pupils may change buses into account. Allowing transfers has several consequences. On the one hand, it allows more flexibility in the bus network structure and can, therefore, help to reduce operating costs. On the other hand, transfers have an impact on the service level: the perceived service quality is lower due to the existence of transfers; however, at the same time, user ride times may be reduced and, thus, transfers may also have a positive impact on service quality. The main objective is the minimization of the total operating costs. We develop a heuristic solution framework to solve this problem and compare it with two solution concepts that do not consider transfers. The impact of transfers on the service level in terms of time loss (or user ride time) and the number of transfers is analyzed. Our results show that allowing transfers reduces total operating costs significantly while average and maximum user ride times are comparable to solutions without transfers. © 2015 Wiley Periodicals, Inc. NETWORKS, Vol. 65(2), 180–203 2015

## 1 INTRODUCTION

In the school year 2011/2012 in Austria (population: 8.42 million 1) about 1.1 million pupils attended one of the 6,120 schools 2. Austria is divided into 121 districts and, on average, each district has 51 schools in total. The number of nonprimary schools ranges from one to 115 with an average of about 25 (across all districts in Austria). Each district consists of multiple municipalities, where the average number of schools in a municipality is 2.57, about half of them being nonprimary schools. If only rural areas are considered, the average number of nonprimary schools per district is 22, the maximum number is 38, and the average number of schools per municipality is about one.

The number of pupils per school differs substantially according to school type and location. Higher level secondary schools are usually located in more densely populated areas whereas primary and secondary schools are also located in rural areas. Primary schools have between 10 and 150 pupils in rural areas. Secondary schools have between 50 and 300 pupils and higher level secondary schools have about 200 to 800 pupils. All of the above data is taken from Statistik Austria 3 and approximated over the whole geographic region of Austria. The low density of schools in rural areas and the distribution of higher level secondary schools require most pupils to use some type of transportation system to get to school.

On the one hand, safety of the pupils during transportation is a crucial factor and must be ensured (i.e., short walking distances, short travel times, we will refer to this as service level). On the other hand, the costs of providing high quality services must be considered by the funding organization (e.g., administration). These two goals are conflicting in nature because high service level often requires dedicated routes for small groups of pupils which require more buses and raises costs.

The pupil transportation system differs from country to country and even from county to county. In some areas, dedicated bus services for every school or group of schools (if they are located close together) are in place. Contrarily, pupil transportation can also be integrated into the public transport system, where pupils use the general public transportation services. Countries often use mixed forms of transportation: In areas where no public transport is available dedicated services are provided and where available the public transportation system must be used. For example, in urban areas with a dense service network pupils may use public transport while in rural areas, dedicated bus services are provided. Other systems provide dedicated bus services for pupils attending primary school, while older pupils have to use public transport.

Austria has a mixed system: in general the public transportation network is used for pupil transportation with the exception of rural areas where dedicated services are provided. For pupils attending primary schools transfers between school buses are not allowed. This means that the planning problem for primary schools can be solved using models without transfers (i.e., approaches proposed in literature, as in 13, 16, 22, 31, 33 can be applied). The transportation network for older pupils can be designed to utilize transfers. In this study, we are interested in the design of the bus transportation network to meet the needs of pupils in secondary schools and older.

Generally, the problem of pupil transportation arises in the morning before the school begins and in the afternoon after school ends. Here, we consider the so‐called morning problem only (i.e., the transportation of the pupils to their school before it begins). The service must be provided only once in the morning, therefore it is nonperiodic. Since the process is the same for every day a feasible solution for a single day can be used during the whole school year.

The overall problem consists of the following subproblems: bus stop selection, bus routing, bus scheduling, and school begin time adjustment. Bus stop selection refers to the process of choosing a proper subset of the set of available bus stops which are then serviced by the bus. This step may also include the assignment of pupils to bus stops. Bus routing is the generation of routes which are serviced by a bus. Typically, bus routes have to respect capacity constraints and often also tour length or duration constraints. Bus scheduling is the computation of a feasible schedule for the buses. It determines which bus route is serviced at which point in time. School begin time adjustment (or bell time adjustment) optimizes the school begin times to allow buses to service multiple schools and thus reduce the number of necessary buses. All these subproblems are strongly interconnected and should be solved in an integrated manner.

Generally, the school bus routing and scheduling problem can be modeled in different ways. If only bus routing and scheduling is considered, and a single school is considered, it can be modeled, for example, as a vehicle routing problem (VRP) 34, where the bus starts at the school, collects the pupils at their bus stops and returns to the school where the pupils are dropped off. In the case where the buses do not start at the school but at the first pickup bus stop, the resulting problem can be modeled as an open vehicle routing problem (OVRP), considering a restriction on the maximum route length. As in case of the (O)VRP all schools are treated independently for every school an OVRP must be solved. An alternative approach is to model pupil transportation as a dial‐a‐ride problem (DARP) where for every pupil a transportation request arises 8. The pickup point is the assigned bus stop of the pupil, the drop off point is the bus stop of the school and pupils of different schools may share a single bus.

A further generalization of the problem is to allow transfers (i.e., pupils attending different schools can share a single bus and can change the bus during their way to school). Transfers may be allowed on a predefined set of bus stops or at arbitrary bus stops. Therefore, the selection of transfer bus stops itself is an optimization problem.

Our contribution is fourfold:

1. Dedicated solution concept for the school bus routing and scheduling problem with transfers, taking into account bus stop selection and pupil assignment, bus routing, and bus scheduling.
2. Evaluation of the solutions considering transfers in terms of costs and service level.
3. Comparison of the solution with transfers with two different modeling approaches without transfers, namely DARP and OVRP.
4. Optimization of the bus stops used for pupil transfers.

In the next section, we give a detailed description of the problem and an overview of the literature. In section 3, the mixed integer linear programming (MILP) model which defines our problem is presented. Then, we describe the heuristic solution concept in detail (section 4). In section 5, we describe how to model the school bus routing and scheduling problem as a DARP and OVRP and describe two different state‐of‐the‐art variable neighborhood search (VNS)‐based solvers, a DARP and an OVRP solver, which are used in section 6. Section 6 gives a detailed analysis of the results of the three approaches by comparing and analyzing the properties of the obtained solutions. Last we summarize our findings and suggest further research directions (section 7).

## 2 PROBLEM STATEMENT AND RELATED WORK

Given is a set *N* of pupils, a set *L* of bus stops, and a set *S* of schools. The bus driving time *t*_*ij*_ between bus stop *i* ∊ *L* and *j* ∊ *L* is given; and also the walking time *u*_*ni*_ of pupil *n* ∊ *N* from her home to bus stop *i* ∊ *L* is known for all pupils. Every pupil *n* has a set of candidate pickup bus stops. The destination bus stop and the school begin time *τ*_*s*_ of every school *s* ∊ *S* as well as the walking times from the destination bus stop to the school are known. The destination school and, therefore, the destination bus stop is known for every pupil *n*.

Additionally, a pupil may arrive at the earliest ${\bar{\omega }}_{s}$ and at the latest ${\underline{\omega }}_{s}$ minutes before school *s* begins. Further, pupils have a minimum $\underline{\gamma }$ and maximum $\bar{\gamma }$ waiting time if they change from one bus to the other.

Every bus *b* ∊ *B* has a maximum capacity *c* which must not be exceeded. It restricts the maximum number of pupils that can be on the bus at the same time. We consider a homogeneous bus fleet.

The objective is to calculate a transportation plan of minimum cost considering the following constraints:

- bus capacity
- upper and lower bounds on waiting times at every school
- upper and lower bounds on waiting times for pupil transfers
- maximum pupil walking time from home to their assigned bus stop

Generally, for a feasible solution the following decision problems must be solved:

1. Assign pupils to bus stops
2. Calculate bus routes
3. Compute pupil routes based on the bus routes
4. Schedule buses to bus routes

**Figure 1:**
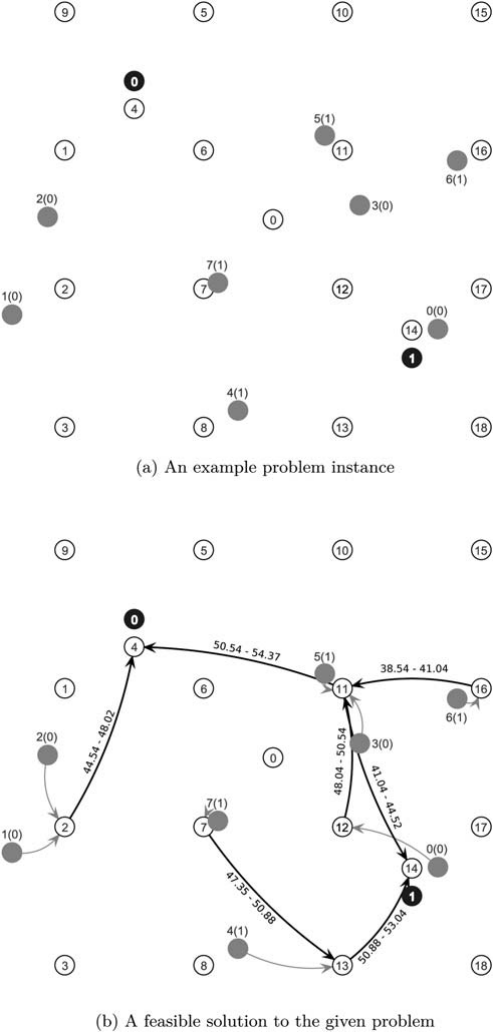
A simple problem instance and a feasible solution.

Pupils with the same pickup and destination bus stop form a single entity and do not split during their ways to school. This is for practical purposes, because in practice it may be difficult to instruct pupils with the same destination waiting at the same pickup bus stop to use different buses. Figure 1a exemplifies a simple problem instance (inst01 from the benchmark set, see section 6). There are 19 bus stops (white circles) numbered from 0 (virtual depot) to 18. Eight pupils (gray) numbered from 0 to 7, the school which they attend is given in parentheses (e.g., pupil five attends school one). Finally, there are two schools (black) numbered 0 and 1. Bus stop 4 is the destination bus stop for school 0 and bus stop 14 is the destination bus stop for school 1. Therefore, pupil 5 must be transported to bus stop 14. Figure 1b shows a feasible solution to the given problem. In the solution, the pupil assignment (arcs without labels), the bus routes (arcs with labels), and the scheduling data (labels on arcs) can be seen. For example, pupil 3 attends school 0 and walks to bus stop 11 to be picked up by a bus. Bus service on arc (11, 4) starts at 50.54 and ends at 54.37, then pupil 3 has to walk to the school. In this example no transfers happen.

The school bus routing and scheduling problem with transfers has not yet been studied extensively in the literature. Newton and Thomas (1969) 20 and Newton and Warren (1970) 21 made one of the first attempts to solve the bus routing problem by the use of a computer for real life cases. They consider bus capacities and maximum user ride time constraints. In their approach, first a giant tour over all bus stops and the school is created. Then, starting from the school they build feasible routes by generating subroutes which satisfy the capacity and ride time constraints and connect them to the school (i.e., every route starts and ends at the school).

Bektaş and Elmastaş (2007) 4 model their real life problem of transporting pupils of an elementary school in Ankara as a capacity and distance constrained OVRP. They minimize the number of buses which represents the operator objective and they use a solver to optimize their MILP model.

Recently, Riera‐Ledesma and Salazar‐González (2012) 28 have proposed a branch‐and‐cut approach for the school bus routing problem with pupil assignment and bus stop selection. They formulate the problem as a multivehicle traveling purchaser problem and discuss their extensions to the classic traveling purchaser model.

Schittekat et al. (2013) 32 investigate the influence of bus stop selection on solution quality. They solve the problem using a parameter free greedy randomized adaptive search procedure combined with a variable neighborhood decent improvement method.

More general approaches do not separate pupils of different schools but allow mixed loads (i.e., the transportation of pupils attending different schools with the same bus). Braca et al. (1997) 6 solve the school bus routing problem with mixed loads for the region of New York City. They construct mixed load routes in a randomized way. Using restarts they are able to generate different routes. Additionally they consider arrival time windows at school and maximum ride times. Park et al. (2012) 24 propose an improvement procedure for mixed loads. First, an initial solution without mixed loads is computed. Then, relocation operators, moving pickup bus stops and, if necessary, also school bus stops, are used to obtain mixed load routes.

Besides the routing problem also the scheduling problem has to be solved. It consists in fixing the departure times of the buses and synchronizing them at transfer points.

Kim et al. (2012) recently proposed a scheduling algorithm for the school bus routing problem without transfers 16. The bus trips to the different schools are considered as given and their duration and the school begin times are known. Buses must then be scheduled so as to transport all pupils to school on time.

Spada et al. (2005) 33 propose a heuristic solution framework for the multiobjective school bus routing and scheduling problem. In their approach, they fix the number of buses and optimize the service level.

A further extension is the consideration of transfers (i.e., pupils may change buses during their way to school). A scheduling model which considers transfers of pupils between buses was proposed by Fügenschuh (2009) 14. He optimizes school begin times to reduce the number of required buses. In the context of the pickup and delivery problem Masson et al. (2012) 18 analyze the effects of transfers on transportation costs. They design an adaptive large neighborhood search algorithm that handles transfers explicitly by special operators. In the context of public transportation, Cortés et al. (2010) 9 propose a branch‐and‐cut solution method for the pickup and delivery problem with transfers. Transfers can only take place at predetermined points, so called transfer nodes. Bouros et al. (2011) 5 also solve the pickup and delivery problem with transfers. They allow transfers at arbitrary points in space, where the detour of two different vehicles is within a certain amount.

An early approach which takes into account school bus routing and scheduling with transfers, pupil assignment, and bell time adjustment was proposed by Desrosiers et al. (1981) 12. They distinguish between urban and rural areas. For rural areas, pupils are collected from their homes and are transported to predefined transfer points, where they change the buses and are transported to their destination school through express routes. For every pupil, it is known to which transfer point she must be transported. In urban areas, pupils are assigned to bus stops where they are picked up by the bus. Routes are generated using modified versions of the Clarke and Wright Savings algorithm 7, Newton's giant tour approach 20 and an insertion approach 29. Then, the routes are scheduled and the school begin times are adjusted so as to minimize the number of buses. They solve the problem hierarchically. The proposed approach does not include a sophisticated solution improvement method.

Recently, a literature overview was published by Park and Kim (2010) 23 which summarizes and categorizes the work in this area.

None of the above concepts explicitly considers the overall problem of bus stop selection, bus routing, bus scheduling, and transfers within a state of the art metaheuristic solution method.

## 3 MATHEMATICAL MODEL

The problem situation considered in this article can be modeled as a MILP, using two types of binary decision variables to determine the bus network and how it is used by the pupils: bus arcs (*x*_*ijb*_) and pupil arcs (*m*_*nijb*_). As the bus arc variables determine the bus lines and each line is served by a single bus, this part of the model is similar to a three‐index VRP formulation, except that a bus stop may be visited multiple times by different buses. The pupil arcs define the path each pupil takes from its home bus stop to the destination bus stop of her school. Pupils can only use those arcs which are serviced by a bus.

For ease of exposition, we introduce an artificial depot denoted by 0 where each bus line must start and end. Each pupil has to be assigned to a single bus stop, where she must be picked up by a bus and use some sequence of bus line arcs until she reaches her destination bus stop (i.e., the bus stop of her school).

Transfers reduce service quality and an excessive number of transfers results in impractical solutions. In our heuristic solution concept, we penalize excessive transfers by an additional term in the objective function. Therefore, we also adhere to this approach in the below model.

In what follows, we first define the different input parameters and then the decision variables we use to model the school bus routing and scheduling problem with transfers.

Input sets:

*N*: set of pupils,
*S*: set of schools,
*L*: set of pickup locations (bus stops), 0 denotes a virtual depot, L′=L∖{0},
*L*_*n*_: set of bus stops pupil *n* may reach walking to start her trip,
*B*: set of available buses.

The following input parameters are used:

*s*_*n*_: school of pupil *n*,
*c*: Bus capacity,
*t*_*ij*_: travel time between *i* ∊ *L*′ and *j* ∊ *L*′,
*e*: earliest point in time when a pupil can be picked up,
*i*_*s*_: bus stop of school *s*,
*τ*_*s*_: begin time of school *s*,
${\bar{\omega }}_{s}$: maximum waiting time at school *s*,
${\underline{\omega }}_{s}$: minimum waiting time at school *s*,
$\underline{\gamma }$: minimum pupil waiting time, when changing from one bus to another,
$\bar{\gamma }$: maximum pupil waiting time, when changing from one bus to another,
*C*: maximum number of transfers per pupil.

We use binary variables,

$$\begin{array}{cc} m_{nijb}=\{\begin{array}{c} 1,\;\text{if pupil}n\text{travels from location}\;i\;\text{to}\;j\;\text{by bus}\;b,\\ 0,\;\text{otherwise}. \end{array}\\ y_{nj}= & \{\begin{array}{c} 1,\;\text{if pupil}\;n\;\text{is assigned}\;\mathrm{to}\;(\text{initial pickup})\;\text{location}\;j,\\ 0,\;\text{otherwise}. \end{array}\\ x_{ijb}= & \{\begin{array}{c} 1,\;\text{if bus}\;b\;\text{travels from}\;i\;\text{to}\;j,\\ 0,\;\text{otherwise}. \end{array}\\ z_{nbi}= & \{\begin{array}{c} 1,\;\text{if bus}\;b\;\text{is left by pupil}\;n\;\text{at bus stop}\;i,\\ 0,\;\text{otherwise}. \end{array}\\ v_{ib^{\prime }b^{\prime \prime }}= & \{\begin{array}{c} 1,\;\text{if at least one pupil changes from bus}\;b^{\prime }\;\text{to}\;b^{\prime \prime }\;\text{at bust stop}\;i,\\ 0,\;\text{otherwise}. \end{array} \end{array}$$

and continuous variables,

*r*_*n*_: number of transfers of pupil *n* exceeding transfer limit *C*,
*A*_*ib*_: arrival time of bus *b* at bus stop *i*,
*T*_*ni*_: arrival time of pupil *n* at bus stop *i*.

The objective function (1) minimizes the total travel time of the buses and it penalizes the number of transfers exceeding the maximum number of allowed transfers *C* (*W* gives the respective penalty term):

(1) $$\min\sum_{i\in L\prime }\sum_{j\in L\prime }\sum_{b\in B}t_{ij}\cdot x_{ijb}+W\cdot \sum_{n\in N}r_{n}.$$

It is subject to a number of constraints. The first set of constraints take care of the assignment of pupils to bus stops. Every pupil *n* must be assigned to a single bus stop *i* out of a set of possible pickup bus stops *L*_*n*_:

(2) $$\sum_{i\in L_{n}}y_{ni}=1,\forall n\in N.$$

The bus line network is defined by constraints (3)–(5). A bus line, if used, must have a single origin at the virtual depot,

(3) $$\sum_{i\in L\prime }x_{0ib}\leq 1,\forall b\in B,$$

the bus line must continue until the virtual depot,

(4) $$\sum_{j\in L,i\neq j}x_{jib}-\sum_{j\in L,i\neq j}x_{ijb}=0,\forall i\in L\prime ,b\in B,$$

and every bus line may only service each bus stop at most once,

(5) $$\sum_{j\in L}x_{ijb}\leq 1,\forall i\in L,b\in B.$$

Based on the bus line network, the paths of the pupils are determined. Pupils may only use arcs that are serviced by the bus line,

(6) $$m_{nijb}\leq x_{ijb},\forall n\in N,i,j\in L\prime ,b\in B.$$

If pupil *n* is assigned to bus stop *i* (*y*_*ni*_ = 1) which is not her destination bus stop $i\neq i_{s_{n}}$ she must leave the pickup bus stop $\left(\sum_{j\in L\prime ,j\neq i}\sum_{b\in B}m_{nijb}=1\right)$. This is ensured as follows:

(7) $$y_{ni}\leq \sum_{j\in L\prime ,j\neq i}\sum_{b\in B}m_{nijb},\forall n\in N,i\in L_{n}|i\neq i_{s_{n}}.$$

Similarly, if pupil *n* arrives at bus stop $h\;(\sum_{i\in L\prime ,i\neq h}\sum_{b\in B}m_{nihb}=1)$ and it is not her destination bus stop $\left(h\neq i_{s_{n}}\right)$, she must travel on to another bus stop $\left(\sum_{j\in L\prime ,j\neq h}\sum_{b\in B}m_{nhjb}=1\right)$:

(8) $$\sum_{i\in L\prime ,i\neq h}\sum_{b\in B}m_{nihb}\leq \sum_{j\in L\prime ,j\neq h}\sum_{b\in B}m_{nhjb},\forall n\in N,h\in L\prime |h\neq i_{s_{n}},$$

(9) $$\sum_{j\in L\prime ,j\neq i}\sum_{b\in B}m_{nijb}\leq 1,\forall n\in N,i\in L\prime .$$

In reality, it is usually impossible to have pupils that attend the same school and are assigned to the same pickup bus stop use different paths in the network. To avoid this situation, we make sure that, if pupil *n*′ travels along arc (*i, j*) and she attends the same school as pupil *n* ^*′′*^ (i.e., $s_{n^{\prime }}=s_{n^{\prime \prime }}$) and leaves from the same pickup bus stop (i.e, $y_{in^{\prime }}=y_{in^{\prime \prime }}=1$), pupil *n* ^*′′*^ uses this arc as well:

(10) $$\sum_{b\in B}m_{n^{\prime }ijb}\leq \sum_{b\in B}m_{n^{\prime \prime }ijb}+(2-y_{hn^{\prime }}-y_{hn^{\prime \prime }}),\forall n^{\prime },n^{\prime \prime }\in N|s_{n^{\prime }}=s_{n^{\prime \prime }},h\in L_{n^{\prime }}\cap L_{n^{\prime \prime }},i,j\in L\prime .$$

We note that pupils *n*′ and *n*″ are not required to use the same bus on this arc; this may not be possible because of capacity restrictions.

The following constraints ensure that only feasible transfers are considered. To make sure that each pupil may only use each bus at most once during her to school journey, we use binary variables *z*_*nbi*_ to indicate whether pupil *n* leaves bus *b* at stop *i*. These variables are set to 1 whenever pupil *n* arrives at bus stop *i* with bus *b*, but does not leave the bus stop with bus *b* ($\sum_{j\in L\prime ,j\neq i}m_{njib}=1$ and $\sum_{j\in L\prime ,j\neq i}m_{nijb}=0$, therefore *z*_*nbi*_ = 1):

(11) $$\sum_{j\in L\prime ,j\neq i}m_{njib}-\sum_{j\in L\prime ,j\neq i}m_{nijb}\leq z_{nbi},\forall n\in N,b\in B,i\in L\prime .$$

Then, to ensure that each pupil *n* may only leave (and thus use) each bus *b* at most once, we use the following set of constraints:

(12) $$\sum_{i\in L\prime }z_{nbi}\leq 1,\forall n\in N,b\in B.$$

To determine if the number of transfers of pupil *n* exceeds the preset transfer limit *C*, we count the number of transfers of pupil *n*. It is given by the number of times pupil *n* leaves a bus at a bus stop that is not her school $\left(\sum_{b\in B}\sum_{i\in L\prime ,i\neq i_{s_{n}}}z_{nbi}\right)$. Variables *r*_*n*_, giving the number of excessive transfers of pupil *n*, are then set as follows:

(13) $$r_{n}\geq \sum_{b\in B}\sum_{i\in L\prime ,i\neq i_{s_{n}}}z_{nbi}-C\forall n\in N.$$

Transfers are only allowed into one direction: either from bus *b*′ to bus *b*″ or from *b*″ to *b*′. This is ensured by two sets of constraints: If at least one pupil changes from bus *b*′ to bus *b*″ at bus stop *i*, then $v_{jb^{\prime }b^{\prime \prime }}=1$,

(14) $$\sum_{i\in L\prime ,i\neq j}m_{nijb^{\prime }}+\sum_{k\in L\prime ,k\neq j}m_{njkb^{\prime \prime }}\leq 1+v_{jb^{\prime }b^{\prime \prime }},\forall j\in L\prime ,n\in N,b^{\prime },b^{\prime \prime }\in B,b^{\prime }\neq b^{\prime \prime },$$

and we make sure that if $v_{jb^{\prime }b^{\prime \prime }}=1$, then $v_{jb^{\prime \prime }b^{\prime }}=0$ and vice versa by means of constraints (15):

(15) $$v_{ib^{\prime }b^{\prime \prime }}+v_{ib^{\prime \prime }b^{\prime }}\leq 1,\forall i\in L\prime ,b^{\prime },b^{\prime \prime }\in B,b^{\prime }<b^{\prime \prime }.$$

The following constraints ensure temporal and logical feasibility. The purpose of these constraints is to synchronize the buses at transfer bus stops, to synchronize pupils and buses, and to ensure time feasibility (i.e., to make sure that pupils arrive at their schools within the respective arrival time windows).

If pupil *n* travels from *i* to *j*, then her arrival time at *i* must be greater or equal to the arrival time at *j* plus the time necessary to travel from *i* to *j*, given by *t*_*ij*_:

(16) $$T_{nj}\geq T_{ni}+t_{ij}-M\cdot \left(1-\sum_{b\in B}m_{nijb}\right),\forall n\in N,i,j\in L\prime .$$

If pupil *n* arrives at bus stop *i* and this is the destination bus stop of school *s* and pupil *n* attends school *s*, then she must arrive $\left(T_{ni_{s_{n}}}\right)$ within the school's arrival time window $\left[\tau_{s}-{\bar{\omega }}_{s},\tau_{s}-{\underline{\omega }}_{s}\right]$. This is ensured by the following constraints:

(17) $$\tau_{s_{n}}-{\bar{\omega }}_{s_{n}}\leq T_{ni_{s_{n}}}\leq \tau_{s_{n}}-{\underline{\omega }}_{s_{n}},\forall n\in N.$$

A bus may not visit a bus stop before the earliest possible time *e*, which, together with the latest possible arrival time at a school $\left(\max_{s}(\tau_{s}-{\underline{\omega }}_{s})\right)$, provides a bound on the travel times (excluding walking times) of the pupils:

(18) $$A_{ib}\geq e,\forall i\in L,b\in B.$$

If bus *b* travels from *i* to *j* then the arrival time *A*_*jb*_ at *j* must be equal to the arrival time at *i* (*A*_*ib*_) plus the respective travel time *t*_*ij*_ (Note that we assume that the service time is included into the travel time):

(19) $$A_{jb}\geq A_{ib}+t_{ij}-M\cdot \left(1-x_{ijb}\right),\forall i\in L,j\in L\prime ,b\in B,$$

(20) $$A_{jb}\leq A_{ib}+t_{ij}+M\cdot \left(1-x_{ijb}\right),\forall i\in L,j\in L\prime ,b\in B.$$

Constraints (21) and (22) make sure that bus and pupil times are synchronized (i.e., if a pupil *n* travels on bus *b*, her arrival time at bus stop *i* has to be equal to the arrival time of bus *b*):

(21) $$T_{ni}\geq A_{ib}-M\cdot \left(1-\sum_{j\in L}m_{njib}\right),\forall n\in N,i\in L,b\in B,$$

(22) $$T_{ni}\leq A_{ib}+M\cdot \left(1-\sum_{j\in L}m_{njib}\right),\forall n\in N,i\in L,b\in B.$$

The following constraints ensure timely synchronization of buses in the case of transfers. If at least one pupil changes from bus *b*′ to bus *b*″ at bus stop *i*, then the arrival time $A_{b^{\prime \prime }}$ of bus *b*″ must not be greater than the arrival time of *b*′ plus the maximum waiting time $\bar{\gamma }$ and not lower than the arrival time of *b*′ plus the minimum waiting time $\underline{\gamma }$.

(23) $$A_{ib^{\prime \prime }}\leq A_{ib^{\prime }}+\bar{\gamma }+M\cdot \left(1-v_{ib^{\prime }b^{\prime \prime }}\right),\forall n\in N,i\in L\prime ,b^{\prime }\in B,b^{\prime \prime }\in B,b^{\prime }\neq b^{\prime \prime },$$

(24) $$A_{ib^{\prime \prime }}\geq A_{ib^{\prime }}+\underline{\gamma }-M\cdot \left(1-v_{ib^{\prime }b^{\prime \prime }}\right),\forall n\in N,i\in L\prime ,b^{\prime }\in B,b^{\prime \prime }\in B,b^{\prime }\neq b^{\prime \prime }.$$

Finally, also bus capacity constraints must be considered. They ensure that on every arc (*i, j*), which is serviced by bus *b*, the number of pupils does not exceed the capacity *c*:

(25) $$\sum_{n\in N,j\in L\prime }m_{nijb}\leq c,\forall i\in L\prime ,b\in B.$$

(26) $$r_{n}\geq 0,\forall n\in N,$$

(27) $$T_{ni}\geq 0,\forall n\in N,i\in L,$$

(28) $$m_{nijb}\in \{0,1\},\forall n\in N,i,j\in L,b\in B,$$

(29) $$y_{ni}\in \{0,1\},\forall n\in N,i\in L_{n},$$

(30) $$x_{ijb}\in \{0,1\},\forall i,j\in L,b\in B,$$

(31) $$v_{ib^{\prime }b^{\prime \prime }}\in \{0,1\},\forall i\in L,b^{\prime },b^{\prime \prime }\in B,b^{\prime }\neq b^{\prime \prime },$$

(32) $$z_{nbl}\in \{0,1\},\forall n\in N,b\in B,l\in L.$$

We use several so‐called big‐M terms. Let *K* denote the latest feasible arrival time at a school across all schools (i.e., $K=\max_{s\in S}\{\tau_{s}-{\underline{\omega }}_{s}\}$). All these terms can conveniently be set to *K*.

The above formulation cannot be solved using state of the art solvers like Gurobi or CPLEX for reasonably sized problems; therefore, we develop a heuristic solution concept.

## 4 HEURISTIC SOLUTION CONCEPT

The idea of the proposed solution concept is to decompose the overall problem into several simpler (hierarchical) subproblems which can be solved in reasonable time by dedicated heuristics, similar to the approach by Desrosiers et al. (1981) 12. However, we include feedback loops that allow information exchange between the different hierarchical levels. If infeasibilities are detected at some level, then this information is conveyed to all earlier stages and, in the next loop, appropriate measures are taken at these earlier stages to avoid the reported infeasibilites at later stages. As destroy and repair (or ruin and recreate)‐based neighborhood search 19, 26 has been successfully applied in the context of several other rich combinatorial optimization problems, we also base our framework on this idea.

Algorithm 1 outlines our solution concept. First a feasible solution is built, which is then improved using a destroy and repair‐based optimization approach 26. To obtain a feasible solution, first, the pupil assignment and bus stop selection problem has to be solved. It determines the bus stops that have to be visited in the bus route generation step. The bus route network, thus, computed in the second step provides the basis for pupil routing (i.e., the identification of the actual path taken by each pupil in the network). This information is again input to the bus scheduling step, determining pupil and bus arrival as well as departure times at the different stops. Our objective is the minimization of the travel costs of the buses (i.e., the sum of the arcs serviced by the buses). The function Cost(s) called in Algorithm 1, line 7 returns the objective value of a solution as defined in (1), where *W* is set to 100 and *C* in Equation (13) is set according to the maximum allowed transfers.

Every step of the algorithm is explained in detail in the following subsections. We first describe the solution construction process (function InitialSolution(input data) in Algorithm 1) and its elements, then we illustrate the neighborhood‐based search method (Algorithm 1, beginning with line 3).

Algorithm 1 **General overview of the solution concept**

1

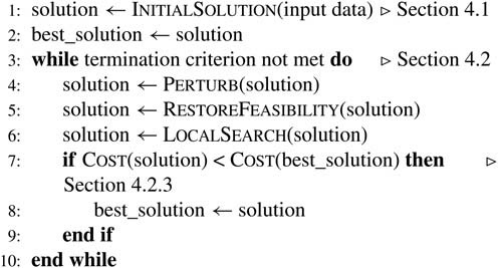

### 4.1 Solution Construction

Algorithm 2 **Solution construction phase**

1

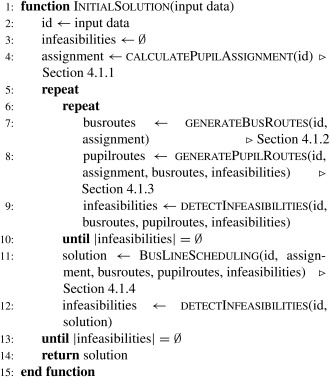

With the exception of pupil assignment and bus stop selection all components are tightly connected. However, it cannot be ensured, that for a given bus routing a feasible pupil routing exists; or in case of a given pupil routing, that a feasible bus scheduling, respecting all temporal constraints, can be determined. Therefore, we exchange information between these three solution construction components. If at any stage an infeasibility is detected, the responsible part of the solution is identified and this information is passed to the earlier stages. There, this information is used to modify the respective solution accordingly.

#### 4.1.1 Pupil Assignment and Bus Stop Selection.

Every pupil must be assigned to a bus stop, where she is picked up by a bus. In our approach, pupil assignment and bus stop selection is done in a single step. We note that, currently, changing the assignment of pupils to departure bus stops is not considered in the optimization. However, to include pupil assignment and bus stop selection into the overall optimization framework, the optimization algorithm is run with several different pupil to bus stop assignment strategies.

We formulate the pupil to bus stop assignment problems as integer programs. Using state‐of‐the‐art commercial solver software, problem instances of realistic size can be solved within a reasonable amount of time.

We use the following three alternative assignment strategies:

##### 4.1.1.1 Minimize Distance to Pupils' Destinations (pa1)

The model is given by objective function (33) and constraints (34), (35), and (36). The objective is to minimize the distance to the pupils' destinations (i.e., pupils are assigned to bus stops which are located in the direction of their destinations).

Consider *h*_*n*_ to be the destination bus stop of pupil *n* and $d_{lh_{n}}$ to be the travel time from bus stop *l* to bus stop *h*_*n*_, then the objective function is:

(33) $$\min\sum_{n\in N}\sum_{l\in L_{n}}d_{lh_{n}}\cdot y_{nl}.$$

It is subject to the following constraints.

Every pupil *n* must be assigned to exactly one bus stop *i*:

(34) $$\sum_{i\in L_{n}}y_{ni}=1,\forall n\in N.$$

The number of pupils that can be assigned to a location *i* is limited by the bus capacity *c*:

(35) $$\sum_{n\in N}y_{ni}\leq c,\forall i\in L,$$

(36) $$y_{ni}\in \{0,1\},\forall n\in N,i\in L.$$

This problem is similar to the capacitated facility location problem (CFLP) 10, whereas the pupils are the customers and the bus stops are facilities. Therefore, this problem may also be solved with special algorithms for the CFLP.

##### 4.1.1.2 Minimize bus stop fragmentation (pa2)

This objective minimizes the number of bus stops where pupils with different destinations are waiting to be picked up. The idea is to assign pupils of different schools to different bus stops and to thus obtain transportation networks for the different schools that are as independent as possible. The model is given by objective function (37) and constraints (34)–(36) and (38)–(41). Let *v*_*is*_ with *i* ∈ *L* and *s* ∈ *S* equal 1 if pupils of school *s* are waiting at bus stop *i*, and 0 otherwise; and let *δ*_*i*_ with *i* ∈ *L* equal 1 if pupils of different schools are waiting at bus stop *i*, 0 otherwise. Constant $M^{\prime }\geq \left|N\right|$, and constant $M^{\prime \prime }\geq \left|S\right|$.

(37) $$\min\sum_{i\in L}\delta_{i}.$$

(38) $$v_{is}\leq \sum_{n\in N|s_{n}=s}y_{ni}\leq M^{\prime }\cdot v_{is},\forall i\in L,\forall s\in S,$$

(39) $$2\cdot \delta_{i}\leq \sum_{s\in S}v_{is}\leq M^{\prime \prime }\cdot \delta_{i}+1,\forall i\in L,$$

(40) $$\delta_{i}\in \{0,1\},\forall i\in L,$$

(41) $$v_{is}\in \{0,1\},\forall i\in L,s\in S.$$

##### 4.1.1.3 Minimize number of bus stops (pa3)

The third model formulation, given by the objective function (42) and constraints (34)–(36), (43), (44) minimizes the number of bus stops used. This choice appears advantageous from the operator perspective: fewer bus stops might result in shorter, and therefore less costly, bus routes. However, the main drawback of this assignment strategy is that there is no information on the pupils' destinations in the assignment phase. This can lead to situations where pupils have to walk long distances into the opposite direction of their school; and this may result in longer travel times and possibly in a higher number of transfer points. Let *q*_*i*_ with *i* ∈ *L* equal 1 if bus stop *i* is used, and 0 otherwise; and constant $M^{\prime \prime \prime }\geq \left|N\right|$.

(42) $$\min\sum_{i\in L}q_{i}.$$

(43) $$q_{i}\leq \sum_{n\in N}y_{ni}\leq M^{\prime \prime \prime }\cdot q_{i},\forall i\in L,$$

(44) $$q_{i}\in \{0,1\},\forall i\in L.$$

All of the above models are solved using IBM ILOG CPLEX, which is fast enough even for large problems. In either case, the solution is an assignment of pupils to bus stops and pupils must be picked up at the selected bus stops. Our bus routing component ensures that this is the case.

#### 4.1.2 Bus Routing.

After the assignment of pupils to bus stops, an initial routing solution is constructed. The bus routing ensures that there is a path for every pupil from the initial pickup bus stop to the destination bus stop. The proposed method is based on the following idea: If we consider only a single school and disregard the capacity constraints of the buses the optimal solution with regard to the objective is a minimum spanning tree (MST): It consists of the shortest edges and connects all nodes which are part of the single school subproblem.

Our approach is the following: For every school *s* a subset of nodes is constructed which contains the bus stops where pupils attending *s* are waiting. Then, we use Prim's algorithm to construct a MST with directed edges for the set of nodes. The starting node is the destination bus stop for school *s*. The edges are directed toward the previous selected bus stop, therefore from every node there is a path to the destination bus stop.

Paths in this tree may become long and, therefore, likely violate time constraints (i.e., they may be longer than the planning horizon). Therefore, we limit the maximum length of a path in the MST. Any path in the spanning tree must be at most as long as the planning horizon of the respective school *s* (i.e., $\tau_{s}-{\underline{\omega }}_{s}-e$).

We achieve the adaptation of the spanning tree by changing the weight matrix which serves for the tree construction. During the tree construction the length of every path from the root node to the current leaves is stored. If the length exceeds the maximum length, the weight matrix is changed in the following way: The weights of the arcs which violate the length restriction and two preceding arcs are increased by a certain amount. Consider the path $\left(p_{n,n-1},\ldots ,p_{t+1,t},p_{t,t-1},p_{t-1,t-2},p_{t-2,t-3},\ldots ,p_{2,1},p_{1,0}\right)$where *p*_0_ is the root node (destination bus stop), *p*_*n*_ is the leave node (bus stop farthest away from school). The path length starts with *e* at the root node and increases along the opposite direction of the path until the leave node. If the travel time exceeds the end of the planning horizon $\left(\tau_{s}-{\underline{\omega }}_{s}\right)$ on this path at arc $p_{t,t-1}$, then the weight for the following arcs is increased: $p_{t,t-1},p_{t-1,t-2},p_{t-2,t-3}$. Using this scheme, long paths in the spanning tree can be shortened. This is repeated until all paths satisfy the maximum length restriction. Weights are only changed temporarily for the construction of the MST.

**Figure 2:**
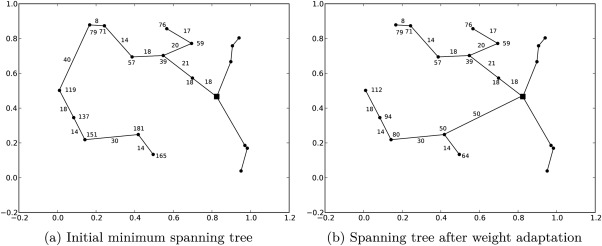
Limiting the length of the paths in the initial bus route graph.

Figure 2 shows an example of a MST based on original weights and a MST based on adapted weights. The circles represent the bus stops which must be visited, the rectangle represents the destination bus stop of the pupils. Arc labels are the length of the arc in the spanning tree and the nodes are labeled according to the cumulative length of the path (starting from the school bus stop). In the example only the relevant subtree is labeled. It can be seen that the longest path in this tree has a length of 165. If we restrict the maximum length of a path in the tree to 120, then the weights are adapted iteratively until every path has a length of at most 120. The final spanning tree is shown in Figure 2b, where the longest path is 112 and thus satisfies the maximum length restriction.

All arcs in the MST are directed toward the respective school. As soon as a feasible bus graph for each school has been found, we obtain a single directed graph by taking the union of all their edges. Therefore, for every pupil a path in this graph exists that starts at her initial bus stop (home) and ends at the destination bus stop (school).

#### 4.1.3 Pupil Routing.

Based on this initial bus route graph, pupil paths are calculated. This is done iteratively for every pupil waiting at a bus stop using a shortest path algorithm on the bus graph taking into account bus capacities (i.e., the arcs are capacitated). As in the previous step, bus capacities were neglected, it is possible and even likely that it is not possible to route all pupils through the graph without violating arc capacities. In this case, the graph has to be augmented.

**Figure 3:**
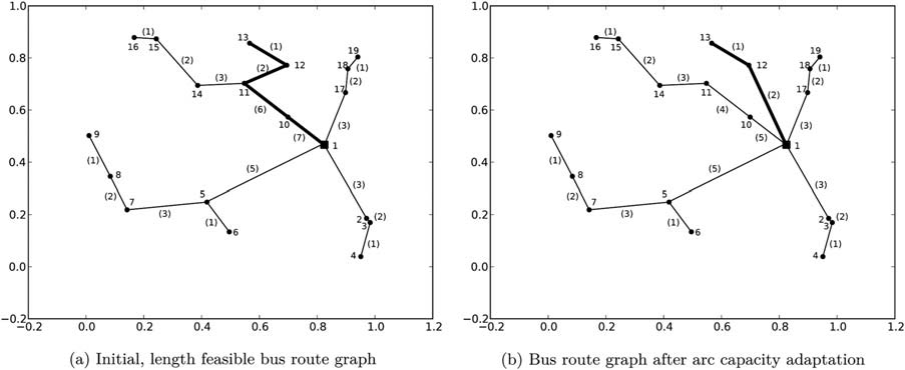
Bus graph augmentation.

Figure 3 shows an example of bus graph augmentation. Figure 3a is the bus graph generated in the previous step with labeled nodes. The labels represent the node numbers. We assume that at every bus stop exactly one pupil is waiting. The arc labels in parenthesis are the arc utilizations (i.e., the number of pupils on the bus servicing this arc). On arc (14, 11) the utilization is 3 and on arc (12, 11) the utilization is 2. Therefore, utilization on arc (11, 10) is 3 + 2 + 1 = 6.

Now assume that the bus capacity respectively arc capacity is 5. On arcs {(11, 10), (10, 1)} this constraint is violated and the graph needs to be adapted.

The idea is to augment the graph as little as possible and, therefore, minimize the additional costs necessary to transport all pupils. For every pupil who does not reach her destination, a set of reachable bus stops is identified. From this set for every bus stop the cost of an arc to the destination bus stop is calculated. The arc with the least cost is added to the bus graph. This is done iteratively until all pupils reach their destination bus stops. The order in which the pupils are considered in the routing is random but consistent (i.e., in every iteration pupils are routed in the same order).

In the example in Figure 3a suppose that pupils waiting at bus stops 13 and 12 are not yet routed through the network. Now the pupil at bus stop 12 is routed. She only reaches bus stop 10, then a capacity constraint violation occurs. Pupil 13 is routed next and reaches bus stop 11 before capacity constraint violation occurs: the residual capacity on arc (11, 10) is zero at that point. For those two pupils, the set of reachable nodes is calculated. For the pupil at node 13 it is {13, 12, 11}. Now the graph is augmented by adding the shortest arc which connects a node from the set of reachable nodes with the destination bus stop. In this example this is arc (12, 1). Figure 3b shows the result with adapted arc utilization.

Now a capacity feasible pupil routing exists and the temporal aspects of this partial solution must be checked. This is done by the bus scheduling component.

#### 4.1.4 Bus Line Scheduling.

The purpose of bus line scheduling is to fix the begin times of the bus routes, such that they are synchronized at the transfer points and pupils reach their schools within the arrival time windows. This synchronization at transfer points possibly leads to waiting times for pupils and buses, and therefore, it may increase the travel time of pupils. Waiting times can only arise at transfer points.

Algorithm 3 shows the general flow of the bus line scheduling.

Algorithm 3 **Bus line scheduling**

1

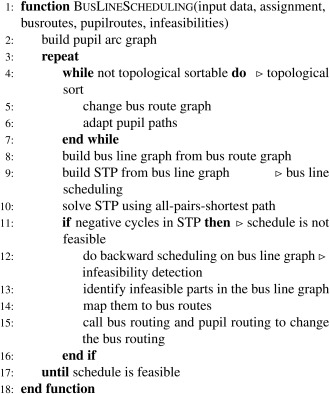

**Figure 4:**
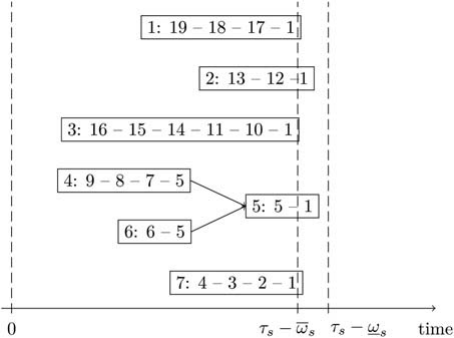
Bus line graph and scheduled bus lines.

It is done on the bus line graph, which is an aggregation of the bus route graph. A bus line is a path in the routing graph where no transfers happen. A simple example based on the routing graph given in Figure 3b is shown in Figure 4. On the *x*‐axis time is drawn where the start of the planning horizon (*e* = 0) and the arrival time window $\left([\tau_{s}-{\bar{\omega }}_{s},\tau_{s}-{\underline{\omega }}_{s}]\right)$ are marked with dashed lines. The rectangles represent the bus lines. For example, bus line 1 visits the bus stops 19, 18, 17, and finally arrives at the school bus stop 1 within the respective arrival time window. In this simple example most bus lines are independent but three bus lines (4, 5, 6) must be synchronized at bus stop 5. For nontrivial problems the bus line graphs have a high number of interdependent bus lines.

**Figure 5:**
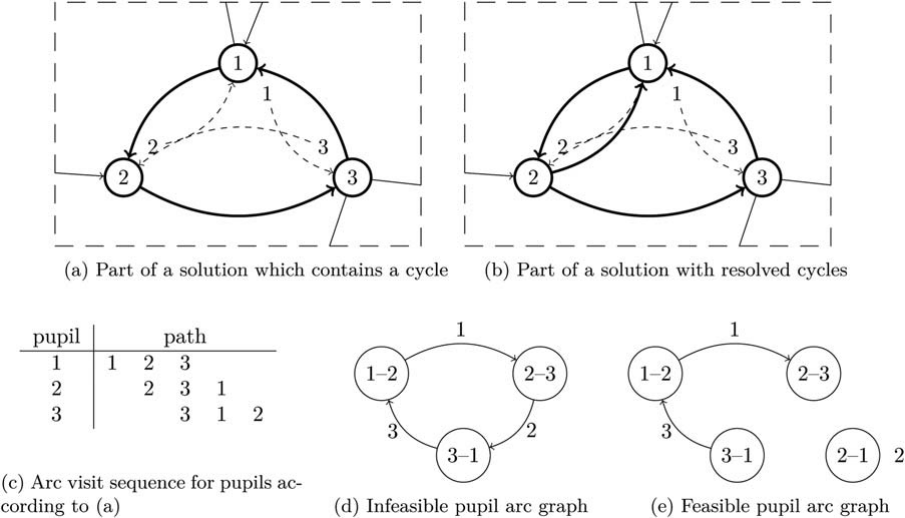
Sequencing of a solution: (a) a cycle in the solution, (b) resolved cycle, (c) temporal order of the pupil paths of the cycle in figure (a), (d) pupil arc graph of cycle depicted in (a), and (e) pupil arc graph of the resolved cycle shown in (b).

Therefore, bus line scheduling is divided into two phases: a preprocessing phase to detect cycles in the solution for which no feasible schedule exists (Algorithm 3, lines 4 – 7), and the scheduling phase. Figure 5 shows an excerpt of a solution which contains such an unresolvable cycle. In this example, pupil 1 is at bus stop 1 and needs to go to bus stop 3 (indicated by the dashed arc), pupil 2 is at bus stop 2 and needs to go to bus stop 1, and pupil 3 is at bus stop 3 and needs to go to bus stop 2. A cycle here is not an arc cycle but a cycle in the sequence of arcs used by pupils. This becomes clear if we add the time dimension as in Figure 5c. There, the pupil paths are aligned according to their arc utilization. And it becomes clear that if arc (1, 2) is serviced first, then arc (2, 3) and finally arc (3, 1), then there is no additional arc which is needed by pupil 3 to get from bus stop 1 to bus stop 2.

The preprocessing detects such situations and repairs them. This is done by building a temporary graph based on the pupil paths (Fig. 5d) and on this graph we apply a topological sort. Every node in the temporary graph is an arc of the bus graph; (1–2) is the arc from bus stop 1 to bus stop 2. The arcs represent a path of a pupil, for example, pupil 1 utilizes arcs (1, 2) and (2, 3). Therefore, an arc [(1 – 2), (2 – 3)] is inserted into the graph. This is done for every pupil. If the resulting graph does not contain a cycle (i.e., it is topologically sortable), an order of arcs exists which can physically be serviced. In this context, we refer to this step as graph sequencing. Even though this could also be integrated into the scheduling, we decided to add an extra step, because the problem of nondecomposable graphs occurs often in case of bigger instances and it is faster to check.

In case the sequencing graph is not topologically sortable (as in Fig. 5d), the pupil routing must be changed and, therefore, the underlying bus graph. To eliminate cycles two different approaches are implemented. The first approach is to determine all arcs which are contained in the cycles and to insert inverted arcs. For example, adding the arc (2,1) to the graph in Figure 5b would eliminate the cycle and result in the pupil arc graph depicted in Figure 5e. The reverse arcs are inserted into the graph and the pupil paths are calculated based on the new graph and the sequencing is done again. This is repeated until the graph is topologically sortable.

If the cycles cannot be removed in this way (i.e., it is detected, that all reversed arcs of the cycle are already in the bus route graph), the strategy is changed. Now shortcuts between two arbitrary nodes in the cycles are added until the graph sequencing is feasible.

Finally, the starting times of the bus routes must be fixed. This is done using the approach of Dechter et al. 11 referred to as STP. They describe an approach where a set of inequalities of the form $a_{1}\leq X_{1}\leq b_{1},a_{2}\leq X_{2}-X_{1}\leq b_{2},\ldots ,a_{n}\leq X_{n}-X_{n-1}\leq b_{n}$ can be transformed into a weighted graph and solved using an all‐pairs shortest path problem. A detailed description of this method is given in 11 and the application in a DARP context is shown in 18. If a solution without negative cycles exists, a feasible schedule exists and the algorithm returns the lower and upper bounds of the variables and fixing those variables to their upper bound yields a feasible solution. In this case, the solution construction process terminates with a feasible solution.

In case there is a cycle in the temporal graph, then no feasible solution exists and the infeasibility must be resolved. It is difficult to determine which component of the solution causes the infeasibility. For example, a trip arrives too late at a school. Is the trip really too long, or is it caused by multiple transfers of pupils and therefore the required synchronization at the transfer points (i.e., induced by waiting time)? Often it is not a single element but the combination of the elements which leads to infeasibilities.

Nonetheless to resolve the infeasibility we use a simple approach. On the temporal aspects of the bus route graph we do a backward scheduling (i.e., we determine the latest times of all routes beginning with the latest arrival time at the schools). Iteratively, we determine the latest times of the preceding events. This allows us to identify the nodes at which the time‐constraint violations occur. Please note that by doing backward scheduling only, we are more restrictive than necessary and may, therefore, exclude feasible solutions.

In case an infeasibility is detected, the bus and pupil routes are analyzed and subpaths which are identified to be part of the infeasibility are stored and must not occur in successive pupil routing attempts. For the bus line graph shown in Figure 4 consider that the waiting time from bus line 6 to bus line 5 at transfer point 5 exceeds the maximum waiting time. Then, the subpath (6, 5, 1) for the pupils is declared forbidden and a different pupil routing on the basis of the bus graph must be found. Forbidden subpaths are stored in a tabu list.

Then, the pupil paths are recalculated under consideration of the forbidden subpaths contained in the tabu list (i.e., those subpaths must not be used on pupil paths). Therefore, the bus route graph must be adapted so that all pupils reach their destination bus stop. This process is repeated until the solution is feasible.

At this point a feasible initial solution is available, but it is likely that the solution utilizes more arcs than necessary. Therefore, arcs which are not necessary are removed from the graph in an iterative manner. Before an arc is removed, it is tested if the solution remains feasible without this arc. In case the arc is necessary for feasibility, it is not removed. Arcs are tested for removal from longest to shortest regardless of the arc utilization. Outgoing arcs of nodes which have only a single outgoing arc are not considered. This way a feasible local optimal solution is generated. Based on this solution the iterative improvement scheme is started.

### 4.2 Neighborhood‐Based Search Method

**Figure 6:**
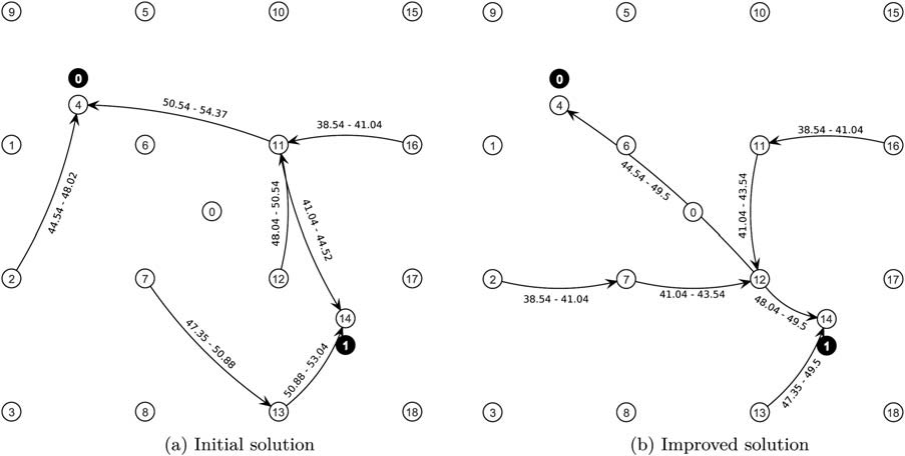
Solution structure comparison of initial solution and improved solution.

Analysis of the solutions after construction showed that in some cases the structure of the initial solution was quite different compared to the optimal routing solution calculated by the solver for small problem instances. Therefore, we designed an improvement method which is able to transform the structure of a given solution in such a way that good solutions can be obtained. Figure 6 emphasizes this by comparing the initial solution and the final, improved solution. It is a problem with two schools, 18 bus stops, and eight pupils. There, it can be seen that the pupil flows of the two solutions are completely different: Figure 6a consists of two independent bus systems, whereas the optimized solution in Figure 6b has a central transfer bus stop from which the pupils are then transported to their destination bus stop. To achieve this, the underlying structure of the bus routes must be changed completely. Conversely, in case the solution is already good and requires only slight modifications to become very good, the optimization method should allow this, too.

We use the idea of destroy and repair‐based neighborhood search 26 for several reasons: First, it currently is state‐of‐the‐art and successfully applied to a number of different, complex routing problems 18, 27, 30. Second, it allows to control the amount of perturbation of a solution by parametrizing the operators, and therefore, it is able to balance exploitation and exploration. Third, additional methods can easily be integrated to adapt to the requirements of slightly different settings (e.g., some new test instances may be hard to optimize utilizing the current operators, then the operators can be adapted easily).

The idea here is to destroy or perturb a solution in terms of structure and then repair this solution in terms of solution quality. In this context, the destroy operator is a solution perturbation and the repair operator is a local search. In Algorithm 1 an outline of our approach is given. After an initial feasible solution is constructed it is iteratively perturbed and reoptimized. The perturbation changes the bus routing graph by removing and inserting arcs without considering arc capacities or temporal constraints. This may result in an infeasible solution and it is necessary to restore feasibility. As we have already developed the methodology to restore feasibility for the solution construction phase the same methods are used in the improvement phase, namely pupil routing and bus line scheduling (see sections 4.1.3 and 4.1.4). Finally, a local search is applied to the perturbed feasible solution. It improves the solution quality by either removing arcs from the bus route graph, or by exchanging long arcs with shorter arcs preserving feasibility.

Hence two different types of operators are needed: perturbation and local search. They are described in the following.

Algorithm 4 **Solution improvement**

1

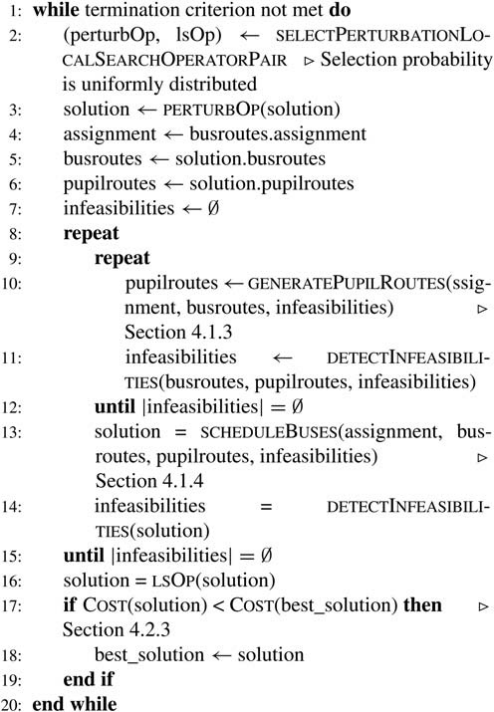

#### 4.2.1 Perturbation.

All operators are randomized and work by deleting and adding arcs in the bus route graph. To prevent cycling, all moves which are induced by an operator are stored in a tabu list. If an arc is removed from the solution, then it must not be added again until it is removed from the tabu list. If an arc is added to the solution, then it must not be removed as long as it is in the tabu list. Two different lists are used, one for forbidden arc removals and one for forbidden arc insertions. The length of the tabu list is a parameter.

For all perturbation operators, the amount how much of the solution is perturbed can be given in percentage of the number of arcs of the solution. It is possible to only slightly change a solution if the amount is low or to change many arcs if the amount is high.

Every perturbation operation consists of two steps: Arc removal, where a certain amount of arcs is removed. Followed by arc insertion, where an operator inserts a certain number of arcs.

**Long arc removal.** All arcs of the graph are sorted according to their length. This list then is used to remove a certain number of arcs. Finally the amount of removed arcs is inserted again randomly, where short arcs are preferred. The amount is either 0.2 *k*, 0.5 *k*, or 0.7 *k*, where *k* gives the number of arcs in the current solution.

**Random arc removal.** A certain amount of random arcs are removed. The same amount of removed arcs are inserted again with a bias toward short arcs. For arc insertion two different variants exist: connect geographically close nodes; do not connect geographically close nodes. The amount is either 0.4 *k*, 0.5 *k*, 0.7 *k*, or 0.9 *k*, where *k* gives the number of arcs in the current solution.

After applying any of the perturbation operators, the solution structure has changed but the new solution is most likely infeasible. Before the local search operator can be applied feasibility must be restored. This is done by applying the pupil routing and bus line scheduling algorithms of the solution construction.

#### 4.2.2 Local Search.

The local search operator improves or repairs a solution by removing arcs from the solution to improve the objective function value in terms of the total length of the bus arcs. It iteratively removes arcs from the current solution. An arc is only removed if the solution stays feasible without the respective arc, else it is not removed. As soon as a valid arc for removal is found, it is removed, (i.e., the arcs are removed in a first‐improvement scheme). The local search continues with the modified solution. We use three different ideas with respect to the order in which arc removals should be tried and performed. They are described in the following.

#### Long arc repair.

This operator tries to remove long arcs in the solution. To achieve this, all arcs are sorted in decreasing order according to their length. Iteratively, every arc is tested for removal with a probability proportional of 0.95^*x*^, where *x* refers to the rank of the arc in the sorted list (i.e., the removal probability decreases geometrically).

#### Residual capacity repair.

This operator tries to remove arcs with low utilization, which are not first in a path. The idea is that arcs with low utilization can likely be removed without a too high solution perturbation in terms of pupil rerouting. The arcs are sorted in decreasing utilization order. Then, iteratively, every arc is tested for removal with a probability proportional of 0.95^*x*^, where *x* refers to the rank of the arc in the sorted list.

#### Random repair.

All arcs are in a random order list and the probability of arc removal for every arc in the list is proportional to 0.95^*x*^, where *x* refers to the rank of the arc in the list.

#### 4.2.3 Solution Evaluation.

The solution evaluation consists of two steps:

1. Determine the length of the bus arcs.
2. Determine the number of transfers for every pupil, in case it is limited.

The length of the solution is determined by summing up the arcs of the solution. However, if the number of maximum allowed transfers is limited, we must determine which bus services which bus lines and if a pupil needs to transfer from one bus to another, see objective function (1) and constraints (13). To determine the number of transfers for the pupils, it is necessary to assign buses to bus lines. The below model determines the minimum number of buses necessary to serve the different bus lines and it returns a feasible schedule for each of the used buses. Based on this result, the number of transfers for pupils is computed, penalized, and added to the solution quality.

Given is the set of bus lines *T*. For every bus line *i* ∈ *T* the start time *A*_*i*_ and its duration *d*_*i*_ is known. The driving time between the last bus stop of line *i* ∈ *T* and the first bus stop of line *j* ∈ *T* is given. Additionally a minimum and a maximum waiting time for a bus which services a consecutive line, $\left[\underline{\theta },\bar{\theta }\right]$, is given.

Based on this information, we are able to identify all time feasible bus line pairs (*i, j*) that can be served consecutively by a single bus. If a bus services line *j* after *i* then the starting time of *j* must be greater then the ending time of *i* plus the service duration of line *i*, given by *d*_*i*_, plus the travel time *t*_*ij*_ to the start location of *j* (plus minimum and maximum waiting time at the beginning of *j*). In case the last stop of *i* is the same as the first stop of *j* (i.e., *t*_*ij*_ = 0), waiting time does not need to be considered. More formally, let *P* denote the set that contains all feasible bus line connections (*i, j*), that is, all those bus line pairs for which one of the following two conditions holds:

(45) $$\text{condition}\;1:\;t_{ij}=0\wedge A_{i}+d_{i}\leq A_{j},$$

(46) $$\text{condition}\;2:\;t_{ij}>0\wedge A_{i}+d_{i}+t_{ij}+\underline{\theta }\leq A_{j}\wedge A_{i}+d_{i}+t_{ij}+\bar{\theta }\geq A_{j}$$

Using this information and binary variables,

$$\begin{array}{c} f_{i}=\{\begin{array}{c} 1,\;\text{if bus line}\;i\;\text{is the first line},\\ 0,\;\text{otherwise}. \end{array}\\ l_{i}=\{\begin{array}{c} 1,\;\text{if bus line}\;i\;\text{is the last line},\\ 0,\;\text{otherwise}. \end{array}\\ c_{ij}=\{\begin{array}{c} 1,\;\text{if bus line}\;i\;\text{is directly followed by bus line}\;j,\\ 0,\;\text{otherwise}. \end{array} \end{array}$$

we are now able to give the integer program that we use to minimize the number of buses. Objective function (47) minimizes the number of first bus lines which corresponds to minimizing the number of buses:

(47) $$\min\sum_{i\in T}f_{i}.$$

It is subject to two sets of constraints. A line *j* is either a first (*f*_*j*_ = 1) line for a bus or has a predecessor $\left(\sum_{i|(i,j)\in P}c_{ij}=1\right)$

(48) $$\sum_{i|(i,j)\in P}c_{ij}+f_{j}=1,\forall j\in T.$$

A line *i* either is the last line (*l*_*i*_ = 1) for a bus or has a successor $\left(\sum_{j|(i,j)\in P}c_{ij}=1\right)$

(49) $$\sum_{j|(i,j)\in P}c_{ij}+l_{i}=1,\forall i\in T.$$

(50) $$f_{i}\in \{0,1\},\forall i\in T,$$

(51) $$l_{i}\in \{0,1\},\forall i\in T,$$

(52) $$c_{ij}\in \{0,1\},\forall (i,j)\in P.$$

## 5 ADDITIONAL MODELING APPROACHES

The school bus routing problem (without transfers) can also be modeled as an OVRP or as a DARP. In case the problem is modeled as an OVRP every school serves as depot and every bus stop at which a pupil for the respective school waits must be serviced by the bus. Pupils waiting at the same bus stop which have the same destination are grouped and treated as a single entity. For every school an independent problem arises and, therefore, multiple OVRP instances must be solved for every school bus routing problem.

The other approach is to model the problem as a DARP. The pupils are pickup up at their initial bus stop and are transported to their destination bus stop. Here, the pupils are treated independently from each other; hence for every pupil a transportation request must be serviced. In case of the DARP pupils of different schools may share a single bus. In this respect, the DARP is more general than the OVRP. Both modeling approaches are described in the following.

### 5.1 Open Vehicle Routing Problem

Given is a complete graph G=(V,E) with the vertex set V={0,1,2,…,n} and the edge set *E*. Vertex 0 is the depot and vertices {1,2,…,n} are the customers. Every customer has a demand *d*_*i*_. The travel time of edge (i,j)∈E is given by *t*_*ij*_, where $t_{ij}\geq 0$. The vehicle capacity of the homogeneous fleet is given by *c*, where $\forall i\in V\setminus \{0\}:d_{i}<c$. In case of the OVRP, the vehicle routes either do not need to start at the depot (in case a pickup problem is given) or do not need to end at a depot (if a delivery problem is given, i.e., $t_{0i}=0$ or $t_{i0}=0,\forall i\in V\setminus \{0\}$). The objective is to find a set of round trips of minimum cost. Every customer must be serviced exactly once with a single visit. For every round trip, the sum of the customer demands must not exceed the vehicle capacity and the length of the round trip must be within an upper bound.

The OVRP solver used here is described in 17 and based on VNS proposed by Mladenović and Hansen 15. The main components of VNS are a set of neighborhood operators, often ordered in increasing neighborhood size, for shaking and a set of operators for local search. Shaking operators perturb a given solution and local search operators improve a solution with regard to the objective function.

At first a feasible solution is perturbed by a shaking operator and then local search is applied to improve the solution. The shaking and improvement cycle is repeated until the search cannot escape from a local optimum. The idea of VNS now is to cycle through different neighborhood operators to perturb the current solution and thus may escape local optima and finally converge to the global optimum.

The VNS used here implements three different shaking operators: cross and icross‐exchange, sequence ruin with reroute heuristic, and random ruin with reroute. Further, the solution concept provides four local search neighborhood operators: 2‐opt, cross‐exchange, or‐opt, 2‐opt*.

For a detailed description of the solution concept and its performance refer to 17.

### 5.2 Dial‐A‐Ride Problem

Given is a complete directed graph G=(V,A). *V* is the set of all vertices and *A* is the set of all arcs. For every arc (*i, j*) a non‐negative travel time *t*_*ij*_ is given. Vehicles are stationed at a depot 0 and must service *n* transportation requests. Each transportation request has an origin *i* and a destination *n* + *i* and a quantity *q*_*i*_, where $q_{n+i}=-q_{i}$. A request (i,n+i) may have a pickup time window $\left[e_{i},l_{i}\right]$ (inbound request) or a delivery time window $\left[e_{n+i},l_{n+i}\right]$ (outbound request). In case of the school bus routing problem all requests have a delivery time window which is determined by the school begin time and the maximum and minimum waiting time at school. Thus, all requests are outbound requests.

Formulating the school bus routing problem as a DARP, allows to transport pupils of different schools in the same vehicle (i.e., mixed loads). Every pupil is a single transportation request and must be picked up at her initial bus stop *i* and must be transported to her destination bus stop *n* + *i*. A time window at the destination bus stop is given that refers to the earliest and latest arrival time at school.

We use the dial‐a‐ride solver proposed in 30 which is based on the work of Parragh et al. 25, it can handle dynamic and stochastic problems. The school bus routing problem is a static problem without stochastic aspects.

The optimization strategy is based on VNS using the following neighborhood operators: move, swap, chain, and zero split. Every operator has an intensity level which ranges from one to five to control the amount of change. For a detailed description please see 30.

## 6 COMPUTATIONAL ANALYSIS

In the previous sections, a solution concept to compute a transportation plan for the school bus routing and scheduling problem with transfers was described, in the following referred to as SBR. Two additional modeling approaches were given. This section summarizes the results obtained by applying the three different solution methods to a set of benchmark problems. We want to answer the following questions:

- How do different modeling and solution techniques perform on different problem instances and what are the differences in the solution properties (i.e., quality and service level)?
- How does the number of maximum allowed transfers influence the solution quality and the service level (time loss and number of transfers)?
- What impact has the pupil assignment strategy on the solution quality and service level?

The quality is defined by the objective function (1) without the penalty term. Therefore, it is the length of the arcs traveled by the buses. The objective in all three alternative modeling approaches (SBR, DARP, and OVRP) is identical. This allows us to compare the results. To measure the service level, we use the time loss of the pupils 33 and the number of transfers. The time loss of a pupil is the difference of the actual travel time from home to school and the shortest travel time (i.e., the pupil is assigned to its nearest bus stop, is picked up by a bus and directly transported to the destination bus stop).

At first we describe the design of the benchmark problems and the setting of the computational experiments (section 6.1). Then an in depth analysis of the results is given (section 6.2) by comparing results of our approach (SBR) to solutions computed by two state‐of‐the art VNS‐based solver, namely a DARP solver 30 and an OVRP solver 17 as described in section 5.

### 6.1 Problem Instances

We use a set of 21 benchmark instances which range from eight pupils and two schools to 500 pupils and eight schools. The benchmarks are generated artificially but are designed to reflect the given situation. As we focus on nonprimary schools, pupils can enrol in the school of their choice and may, therefore, be located in the whole geographic area. The bus stops have the same geographic location in all instances, whereas pupil home locations vary. The planning horizon starts at zero (i.e., *e* = 0) and school begin times are 60 min. Therefore, the planning horizon is an hour.

Table 1 summarizes the general properties of the problem instances. For every row in this table the instances differ only in the pupil location, all other parameters are fixed.

**Table 1:** Overview of the problem instance properties.

Instance	Pupils	Schools	Bus capacity
inst01 – inst03	8	2	4
inst04 – inst05	100	2	40
inst06 – inst07	200	2	40
inst08 – inst09	500	2	40
inst10 – inst11	100	4	40
inst12 – inst13	200	4	40
inst14 – inst15	500	4	40
inst16 – inst17	100	8	40
inst18 – inst19	200	8	40
inst20 – inst21	500	8	40

The bounds on the arrival time at school *s* are ${\underline{\omega }}_{s}=5,\;{\bar{\omega }}_{s}=40,\;\forall s\in S$, and the bounds on the bus change waiting time are $\underline{\gamma }=1$, and, $\bar{\gamma }=10$ in all instances. A time window is defined, which specifies the minimum $\left(\underline{\theta }\right)$ and maximum $\left(\bar{\theta }\right)$ allowed waiting time for a bus if it services another bus line after the current one. These parameters are: $\underline{\theta }=0$ and $\bar{\theta }=30$.

### 6.2 Analysis

All of the following computations were performed on an Intel Core i7‐3770 CPU with 3.40 GHz with 16GB RAM and a maximum runtime of 1 h. The solution concept was implemented in Java and executed using the Java SE Runtime Environmnent 1.8.0 and the HotSpot 64‐Bit Server VM. The runtime for every instance is 60 min. For all approaches pupils are routed in groups (i.e., pupils which have the same pickup bus stop and the same destination bus stop are routed as a single entity). In the following, we give summary based on the results given in Tables 8, 9, 10, 11, 12, 13, 14, 15, 16, 17, 18, 19. Solution quality and time loss are given in minutes.

6.2.1

##### How do different modeling and solution techniques perform on different problem instances and what are the differences in the solution properties (i.e., quality and service level)?

In Table 2, we provide average results for the three different modeling approaches considering the proposed benchmark instances which contain multiple schools. For these experiments, we use pupil assignment strategy pa1 (i.e., we minimize the distance to the pupils' destination bus stop when determining the starting bus stop). Furthermore, more than one transfer per pupil is penalized (i.e., *C* = 1, *W* = 100, where *C* is the number of maximum allowed transfers and *W* refers to the penalty weight). We note again that the other two approaches do not allow transfers. On average, the solution quality obtained by means of the OVRP solver (127.997 min) is 66.3% worse and the solution quality of the DARP solver (62.339 min) is 18.9% better compared to the solution quality obtained by the proposed SBR approach (76.947 min). The average time loss of the OVRP solutions is 5.093 min and the time loss of the DARP solutions is 9.422 min, that is, 3.98% respectively 92.3% worse compared to the time loss of the SBR solutions (4.898 min). The good solution quality of the DARP solver comes at the cost of a high average time loss for the pupils. The comparison of instances which yield similar solution quality between SBR and DARP, (e.g., two schools, 100 and 200 pupils; eight schools, 100 and 200 pupils) shows that transfers allow a lower average time loss of the pupils. The solution quality of the OVRP approach deteriorates with an increasing number of schools, because for every school an OVRP is solved. Therefore, the solutions for the schools are independent (i.e., pupils of different schools cannot be on the same bus) and a lot of arcs are visited multiple times by buses for different schools. For the SBR approach, the average number of transfers per pupil ranges from 0.252 to 0.535 (i.e., 25% – 53% of the pupils must change the bus on their way to school), for instances with at least 100 pupils. Over all instances the average transfers of pupils is 0.377 (i.e., nearly 38% of the pupils must change the bus one time). Detailed results are in Tables 10 and 11.

**Table 2:** Comparison of solution quality and service level for different approaches, pupil assignment strategy minimize distance to pupils' destinations (pa1), and at most one transfer.

		Quality (avg)			Time loss (avg)			Transfers (avg)
Schools	Pupils	DARP	OVRP	SBR	DARP	OVRP	SBR	SBR
2	8	19.081	19.314	**17.967**	5.053	**3.736**	5.499	0.167
2	100	54.137	69.957	**47.430**	8.954	5.653	**5.569**	0.360
	200	**47.887**	74.439	51.778	10.787	**4.048**	4.207	0.315
	500	**49.004**	91.907	76.992	10.040	3.646	**3.234**	0.252
4	100	68.226	116.067	**62.061**	10.115	5.699	**4.836**	0.435
	200	70.920	141.471	**62.197**	9.394	5.843	**5.745**	0.425
	500	**73.354**	157.309	96.849	10.046	**3.666**	4.531	0.408
8	100	**74.155**	183.006	79.872	9.043	5.565	**5.193**	0.535
	200	69.594	131.865	**63.905**	10.647	5.742	**4.422**	0.458
	500	**97.028**	294.634	210.419	10.138	7.328	**5.743**	0.418
average		**62.339**	127.997	76.947	9.422	5.093	**4.898**	0.377

Tables 3 and 4 show the results for the two other pupil assignment strategies (i.e., minimizing bus stop fragmentation [pa2] and minimizing the number of bus stops [pa3]). Here, the average time loss of the pupils increases for all three approaches, and the objective function value decreases. The pupil assignment strategy influences the solution quality and the time loss of the pupils. A better solution quality implies a higher pupil time loss. For the SBR approach, pupil assignment strategies 2 and 3 also lead to a higher number of average transfers compared to pupil assignment strategy 1.

**Table 3:** Comparison of solution quality and service level for different approaches, pupil assignment strategy minimize bus stop fragmentation (pa2), and at most one transfer.

		Quality (avg)			Time loss (avg)			Transfers (avg)
Schools	Pupils	DARP	OVRP	SBR	DARP	OVRP	SBR	SBR
2	8	20.081	19.265	**18.592**	4.465	**3.365**	3.751	0.167
2	100	44.143	63.739	**38.538**	13.664	7.382	**5.364**	0.410
	200	**43.923**	71.174	53.060	13.271	4.872	**4.871**	0.535
	500	**49.177**	85.725	79.658	11.092	**4.030**	4.455	0.299
4	100	**48.439**	117.298	54.116	10.284	8.649	**5.287**	0.515
	200	**61.068**	137.237	61.332	12.732	7.036	**6.304**	0.545
	500	**76.917**	159.128	101.897	11.040	**4.604**	5.099	0.444
8	100	**66.520**	189.352	84.059	10.967	**6.511**	7.916	0.605
	200	**61.195**	129.522	66.008	12.748	6.931	**5.267**	0.430
	500	**73.483**	282.943	174.297	10.491	7.247	**6.217**	0.519
average		**54.495**	125.538	73.156	11.075	6.063	**5.453**	0.447

**Table 4:** Comparison of solution quality and service level for different approaches, pupil assignment strategy minimize number of bus stops (pa3), and at most one transfer.

		Quality (avg)			Time loss (avg)			Transfers (avg)
Schools	Pupils	DARP	OVRP	SBR	DARP	OVRP	SBR	SBR
2	8	**18.227**	19.247	19.247	4.299	3.112	**2.710**	0.083
2	100	43.070	63.739	**39.200**	11.496	7.382	**5.186**	0.390
	200	**47.375**	71.174	52.916	15.192	**4.872**	4.916	0.525
	500	**49.025**	85.725	80.447	11.740	**4.030**	4.325	0.301
4	100	**55.480**	117.298	55.817	13.074	8.649	**5.247**	0.510
	200	66.715	137.237	**61.722**	12.766	7.036	**5.776**	0.570
	500	**74.146**	159.128	101.255	11.738	**4.604**	5.007	0.428
8	100	**70.559**	189.352	94.464	12.619	**6.511**	6.852	0.475
	200	68.011	129.522	**61.594**	12.121	6.931	**6.774**	0.475
	500	**94.746**	282.943	134.046	11.516	**7.247**	8.018	0.564
average		**58.735**	125.536	70.071	11.656	6.037	**5.481**	0.432

The comparison of solution quality for different pupil assignment strategies for the SBR approach shows that the quality using pupil assignment strategy pa1 (minimize distance to pupils' destinations) is worse compared to the other two assignment strategies. Pupil assignment strategies pa2 and pa3 lead to better solution quality, because they tend to utilize a lower number of bus stations and, therefore, need to visit fewer stops. This is obvious for strategy pa3 (minimize number of bus stops) but also true in the case of strategy pa2 (minimize bus stop fragmentation). The reason is that pupils for different schools are distributed in the whole geographic area. Therefore, it is often not possible to assign pupils to a bus stop so that only pupils of a single school are waiting there. This indirectly leads to minimizing the number of used bus stops, because the objective function minimizes the number of fragmented bus stops. In case most or all bus stops are fragmented, it leads to minimization of the number of bus stops. However, the effect strongly depends on the geographical distribution of the pupils. Averaged over all instances, assignment strategy pa1 uses 91% of the bus stops (i.e., in almost all cases, except for small instances, all bus stops are used); whereas assignment strategies pa2 and pa3 utilize only about 76% of the bus stops. Due to the lower number of used bus stops, pupil travel path lengths increase on average and, therefore, also the time loss. The detailed results for pa2 (minimizing bus stop fragmentation) are in Tables 14 and 15. For pupil assignment strategy pa3 (minimizing number of bus stops) the results can be found in Tables 18 and 19.

Table 5 compares the results of the DARP, OVRP, and SBR approach for an unlimited number of allowed transfers in case of the SBR. Here, we see that on average the DARP (62.339) and SBR (62.606) results are comparable in terms of solution quality. However, the time loss is 9.422 for the DARP results and 5.133 for the SBR results, with an average of 0.556 transfers per pupil. The maximum number of transfers for those instances is three (see Table 8).

**Table 5:** Comparison of solution quality and service level for different approaches, pupil assignment strategy minimize distance to pupils' destinations (pa1), and unlimited number of transfers.

		Quality (avg)			Time loss (avg)			Transfers (avg)
Schools	Pupils	DARP	OVRP	SBR	DARP	OVRP	SBR	SBR
2	8	19.081	19.314	**17.967**	5.053	**3.736**	5.499	0.167
	100	54.137	69.957	**46.939**	8.954	5.653	**5.443**	0.490
	200	**47.887**	74.439	52.347	10.787	**4.048**	4.183	0.348
	500	**49.004**	91.907	77.293	10.040	3.646	**3.181**	0.259
4	100	68.226	116.067	**53.573**	10.115	**5.699**	6.210	0.825
	200	70.920	141.471	**57.059**	9.394	5.843	**4.869**	0.592
	500	**73.354**	157.309	93.762	10.046	**3.666**	5.096	0.526
8	100	74.155	183.006	**64.189**	9.043	**5.565**	6.477	0.995
	200	69.594	131.865	**57.178**	10.647	**5.742**	4.006	0.602
	500	**97.028**	294.634	105.753	10.138	7.328	**6.366**	0.752
average		**62.339**	127.997	62.606	9.422	**5.093**	5.133	0.556

**Figure 7:**
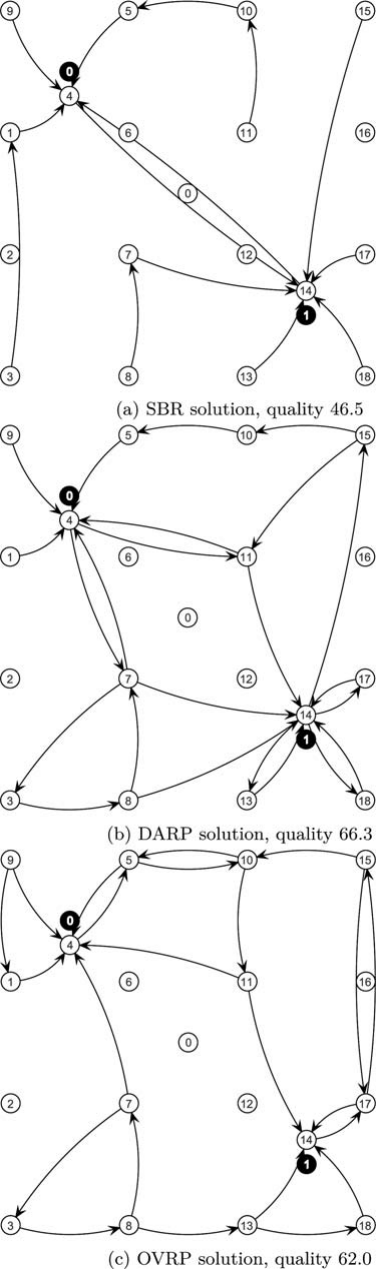
Comparison of solution structure of the different modeling approaches.

We also compare the structure of the solutions calculated with the different solution techniques. Figure 7 compares solutions of the same problem instance (inst07, bus capacity 20) for the three approaches. The symmetry of the solution in case of the SBR approach can easily be seen (Fig. 7a). First the pupils are transfered to a central transfer point, one on the upper left (4) and one on the lower right (14). There they possibly change buses and are then transported to their destination school. In comparison, in the solutions computed by the other two approaches (Fig. 7b and c) some bus stops have to be visited multiple times. Additionally, some arcs are traversed multiple times (in different directions).

##### How does the number of maximum allowed transfers influence the solution quality and the service level (time loss and number of transfers)?.

Table 6 shows the results averaged over all test instances with at least 100 pupils. The column max. transfers refers to the number of maximum allowed transfers, and the column quality, time loss, transfers refer to the respective solution property. For every property mean and maximum are given. We see that the solution quality improves with the number of maximum allowed transfers. If only one transfer is permitted the mean of the solution quality is 79.479, which is 17.9% higher than with unlimited number of allowed transfers (67.406). However, the average number of transfers is 0.45 if at most one transfer is allowed, whereas the average number of transfers is 0.639 in case of an unlimited number of transfers (i.e., for an unlimited number of transfers the average number of transfers per pupil is 42% higher than in the case where only at most one transfer per pupil is allowed). The difference in time loss is about 6% (at most one transfer [5.421] versus unlimited number of transfers [5.806]). From this table, we see that, as the number of allowed transfers increases, the solution quality improves, while the service level deteriorates. Especially the improvement from allowing two transfers instead of one transfer is high. This is due to the structure of the test instances. For most instances, allowing more than two transfers per pupil does not allow to find solutions of improved quality. Only in very few instances some pupils utilize three transfers, when the number of allowed transfers is unlimited, and only in a single case at most four transfers per pupil are used (inst17, Table 16). The numbers for this analysis are in Tables 8, 9, 10, 12, 13, 14, and 16, 17, 18.

**Table 6:** Solution quality, number of transfers, and time loss over all instances with at least 100 pupils.

	Quality		Time loss		Transfers	
Max. transfers	Mean	Maximum	Mean	Maximum	Mean	Maximum
1	79.479	243.259	5.421	38.265	0.450	1
2	68.464	117.017	5.552	37.236	0.602	2
unlimited	67.406	110.443	5.806	37.360	0.639	4

##### What impact has the pupil assignment strategy on the solution quality and the service level?

Table 7 compares solution quality and service level for different pupil assignment strategies and maximum allowed number of transfers. Again, only instances with at least 100 pupils are considered. Therefore, the numbers are slightly higher than in Tables 2, 3, 4. In the cases, where the maximum number of transfers is 1, the solution quality for different assignment strategies varies in the range of 75.72 – 83.50, which is about 10% difference. In the case of an unlimited number of allowed transfers the range of variation is 67.324 – 67.566, which is about 0.36%. We see that by allowing transfers the achievable solution quality increases and the solution quality range decreases. This, however, comes with an increase in the time loss as well as the number of transfers, which can also be seen in Table 7. For pupil assignment strategy pa1 (minimize distance to pupils' destinations), where the improvement in solution quality is highest, the time loss increases from 4.831 to 5.092 (about 5.4%) and the number of transfers increases from 0.401 to 0.599 (about 49.4%). For pupil assignment strategy pa2 (minimize bus stop fragmentation) those increases are from 5.642 to 6.342 (12.1%) and from 0.478 to 0.672 (40%) for the average time loss respectively transfers. The increase in time loss is 3.4% (from 5.789 to 5.984) and the increase in the number of transfers is 37% (from 0.471 to 0.648) for the pupil assignment strategy minimizing the number of bus stops (pa3). As expected, a higher improvement in solution quality implies a higher deterioration in the service level. Also, the improvement in solution quality from at most two transfers to an unlimited number of allowed transfers is small due to the fact that at most four transfers occur over all test cases, see Table 16, inst17. So, allowing transfers can compensate for bad pupil assignment in terms of solution quality at the cost of a lower service level. Detailed results are in Tables 8, 9, 10, 12, 13, 14, and 16, 17, 18.

**Table 7:** Solution quality, service level, and assignment strategy for maximum 1, 2 or unlimited transfers over all instances with at least 100 pupils.

	Quality (avg)			Time loss (avg)			Transfers (avg)		
Assignment	1	2	3	1	2	3	1	2	3
No. of tranfers									
1	83.500	79.218	**75.718**	**4.831**	5.642	5.789	**0.401**	0.478	0.471
2	**68.149**	68.706	68.537	**4.804**	5.843	6.009	**0.536**	0.634	0.637
unlimited	67.566	**67.324**	67.329	**5.092**	6.342	5.984	**0.599**	0.672	0.648

**Table 8:** Solution quality of different problem instances and pupil assignment strategy 1 and unlimited pupil transfers.

			Time loss	Time loss	Transfers	Transfers
Instance	Quality	Buses	(max)	(avg)	(max)	(avg)
inst01	19.933	3	17.534	7.160	1	0.250
inst02	19.933	3	19.998	8.205	1	0.250
inst03	14.035	2	4.456	1.134	0	0.000
inst04	46.939	7	22.978	5.444	1	0.320
inst05	46.939	6	23.264	5.441	2	0.660
inst06	54.166	10	18.881	4.572	1	0.455
inst07	50.528	9	18.553	3.794	1	0.240
inst08	77.806	12	19.734	3.015	1	0.202
inst09	76.781	12	23.456	3.346	1	0.316
inst10	51.593	9	23.912	6.059	3	0.890
inst11	55.553	10	24.297	6.362	3	0.760
inst12	58.538	10	18.175	4.518	2	0.500
inst13	55.581	9	21.346	5.219	2	0.685
inst14	96.212	9	26.118	5.039	3	0.562
inst15	91.311	12	21.089	5.154	2	0.490
inst16	63.052	7	20.622	6.207	3	0.870
inst17	65.327	9	21.707	6.747	3	1.120
inst18	55.634	9	18.382	4.217	3	0.575
inst19	58.722	10	18.764	3.795	2	0.630
inst20	106.988	9	37.360	7.031	2	0.666
inst21	104.518	10	28.634	5.700	3	0.838

**Table 9:** Solution quality of different problem instances and pupil assignment strategy 1 and at most two pupil transfers.

			Time loss	Time loss	Transfers	Transfers
Instance	Quality	Buses	(max)	(avg)	(max)	(avg)
inst01	19.930	3	17.534	7.160	1	0.250
inst02	19.930	3	19.998	8.205	1	0.250
inst03	14.040	2	4.456	1.134	0	0.000
inst04	46.940	7	22.978	5.444	2	0.530
inst05	46.940	6	23.264	5.441	2	0.660
inst06	53.030	10	21.688	4.619	1	0.390
inst07	50.530	9	18.553	3.794	1	0.240
inst08	78.570	12	15.478	2.699	1	0.320
inst09	77.810	12	20.691	3.373	1	0.310
inst10	52.180	10	17.231	4.972	2	0.560
inst11	52.180	10	16.310	4.716	2	0.580
inst12	58.880	10	18.175	4.659	2	0.495
inst13	57.600	9	21.346	5.171	2	0.605
inst14	92.930	11	22.357	4.391	2	0.500
inst15	91.830	10	34.774	5.036	2	0.518
inst16	69.130	8	24.478	5.460	2	0.760
inst17	68.790	11	19.803	6.430	2	0.780
inst18	56.300	10	17.936	3.886	2	0.545
inst19	58.720	10	18.764	3.849	2	0.630
inst20	104.930	10	28.069	6.454	2	0.644
inst21	109.400	11	27.524	6.070	2	0.574

**Table 10:** Solution quality of different problem instances and pupil assignment strategy 1 and at most one pupil transfer.

	DARP				OVRP			
Instance	Quality	Buses	Time loss (max)	Time loss (avg)	Quality	Buses	Time loss (max)	Time loss (avg)
inst01	21.950	4	13.900	4.094	21.950	4	13.901	3.615
inst02	21.257	2	21.778	7.149	21.957	3	13.359	5.326
inst03	14.035	2	12.421	3.917	14.035	2	4.456	2.268
inst04	58.282	5	22.804	9.503	74.499	7	26.087	5.486
inst05	49.992	3	27.913	8.404	65.415	7	24.357	5.820
inst06	51.721	3	39.949	10.563	76.246	9	15.785	4.223
inst07	44.052	4	40.583	11.012	72.632	9	18.848	3.874
inst08	49.004	3	36.881	9.638	92.147	10	19.163	3.462
inst09	49.004	3	37.459	10.442	91.668	10	17.708	3.830
inst10	63.481	4	34.606	12.041	114.167	8	21.733	7.321
inst11	72.970	2	34.516	8.188	117.968	13	18.140	4.076
inst12	74.967	4	36.634	9.309	145.812	12	28.381	6.520
inst13	66.873	4	38.129	9.479	137.131	12	22.776	5.166
inst14	72.691	4	42.085	9.985	157.344	16	20.164	3.696
inst15	74.016	3	41.070	10.107	157.274	16	17.460	3.636
inst16	67.909	2	29.680	8.680	171.580	15	26.258	6.533
inst17	80.401	3	33.923	9.407	194.431	20	24.995	4.597
inst18	67.190	3	33.704	10.879	133.487	12	28.660	6.507
inst19	71.999	4	37.168	10.416	130.244	12	18.848	4.978
inst20	109.280	4	39.336	9.951	297.935	23	29.070	7.592
inst21	84.776	2	39.286	10.325	291.332	22	27.506	7.065

**Table 11:** Solution quality of the problem instances and pupil assignment strategy 1 for the DARP solver and the OVRP solver.

	DARP				OVRP			
Instance	Quality	Buses	Time loss (max)	Time loss (avg)	Quality	Buses	Time loss (max)	Time loss (avg)
inst01	21.950	4	13.900	4.094	21.950	4	13.901	3.615
inst02	21.257	2	21.778	7.149	21.957	3	13.359	5.326
inst03	14.035	2	12.421	3.917	14.035	2	4.456	2.268
inst04	58.282	5	22.804	9.503	74.499	7	26.087	5.486
inst05	49.992	3	27.913	8.404	65.415	7	24.357	5.820
inst06	51.721	3	39.949	10.563	76.246	9	15.785	4.223
inst07	44.052	4	40.583	11.012	72.632	9	18.848	3.874
inst08	49.004	3	36.881	9.638	92.147	10	19.163	3.462
inst09	49.004	3	37.459	10.442	91.668	10	17.708	3.830
inst10	63.481	4	34.606	12.041	114.167	8	21.733	7.321
inst11	72.970	2	34.516	8.188	117.968	13	18.140	4.076
inst12	74.967	4	36.634	9.309	145.812	12	28.381	6.520
inst13	66.873	4	38.129	9.479	137.131	12	22.776	5.166
inst14	72.691	4	42.085	9.985	157.344	16	20.164	3.696
inst15	74.016	3	41.070	10.107	157.274	16	17.460	3.636
inst16	67.909	2	29.680	8.680	171.580	15	26.258	6.533
inst17	80.401	3	33.923	9.407	194.431	20	24.995	4.597
inst18	67.190	3	33.704	10.879	133.487	12	28.660	6.507
inst19	71.999	4	37.168	10.416	130.244	12	18.848	4.978
inst20	109.280	4	39.336	9.951	297.935	23	29.070	7.592
inst21	84.776	2	39.286	10.325	291.332	22	27.506	7.065

**Table 12:** Solution quality of different problem instances and pupil assignment strategy 2 and unlimited pupil transfers.

			Time loss	Time loss	Transfers	Transfers
Instance	Quality	Buses	(max)	(avg)	(max)	(avg)
inst01	20.842	3	12.969	4.982	1	0.500
inst02	21.016	3	11.155	4.528	0	0.000
inst03	13.919	3	9.871	1.742	0	0.000
inst04	40.643	7	25.670	5.946	1	0.490
inst05	36.776	6	18.555	4.790	1	0.260
inst06	53.358	11	21.952	5.617	1	0.620
inst07	50.605	8	18.686	4.803	1	0.350
inst08	79.610	12	20.309	4.450	1	0.276
inst09	79.331	11	23.488	5.277	2	0.396
inst10	44.516	7	22.077	6.991	3	0.940
inst11	54.845	9	25.215	6.198	2	0.660
inst12	58.900	9	22.211	5.618	3	0.810
inst13	58.611	9	22.623	5.653	2	0.875
inst14	102.281	10	31.962	6.360	2	0.608
inst15	90.820	10	25.685	5.503	3	0.586
inst16	57.522	6	31.319	11.916	2	1.030
inst17	69.271	9	28.461	9.984	3	1.090
inst18	59.935	10	19.199	4.690	3	0.745
inst19	57.712	9	24.843	6.123	3	0.860
inst20	108.904	11	29.296	7.223	3	0.802
inst21	108.190	8	36.404	7.004	2	0.690

**Table 13:** Solution quality of different problem instances and pupil assignment strategy 2 and at most two pupil transfers.

			Time loss	Time loss	Transfers	Transfers
Instance	Quality	Buses	(max)	(avg)	(max)	(avg)
inst01	20.842	3	12.969	4.982	1	0.500
inst02	21.016	3	11.155	4.528	0	0.000
inst03	13.919	3	9.871	1.742	0	0.000
inst04	41.939	8	25.803	5.876	1	0.390
inst05	36.776	6	19.091	4.783	2	0.370
inst06	53.208	12	18.369	4.826	1	0.585
inst07	52.911	8	18.553	4.916	1	0.485
inst08	81.254	12	21.355	4.616	1	0.308
inst09	80.890	10	26.213	5.087	2	0.332
inst10	50.515	9	22.197	6.902	2	1.050
inst11	54.845	10	25.215	5.887	2	0.810
inst12	56.220	7	27.531	6.158	2	0.635
inst13	60.043	9	25.258	5.306	2	0.550
inst14	99.379	13	25.504	4.819	2	0.520
inst15	97.690	12	25.682	5.285	2	0.556
inst16	59.539	6	26.319	8.877	2	0.910
inst17	70.442	7	33.112	8.207	2	0.980
inst18	58.357	9	20.310	5.065	2	0.700
inst19	60.807	10	20.906	4.452	2	0.745
inst20	106.630	9	31.837	6.812	2	0.674
inst21	115.266	11	33.543	7.293	2	0.820

**Table 14:** Solution quality of different problem instances and pupil assignment strategy 2 and at most one pupil transfer.

			Time loss	Time loss	Transfers	Transfers
Instance	Quality	Buses	(max)	(avg)	(max)	(avg)
inst01	20.842	3	12.969	4.982	1	0.500
inst02	21.016	3	11.155	4.528	0	0.000
inst03	13.919	3	9.871	1.742	0	0.000
inst04	40.301	8	24.767	5.370	1	0.410
inst05	36.776	6	19.091	5.359	1	0.410
inst06	53.208	12	18.369	4.826	1	0.585
inst07	52.911	8	18.553	4.916	1	0.485
inst08	79.640	13	20.309	4.298	1	0.328
inst09	79.677	11	22.628	4.613	1	0.270
inst10	51.611	7	22.771	5.283	1	0.520
inst11	56.621	6	19.262	5.291	1	0.510
inst12	59.447	8	29.283	5.999	1	0.490
inst13	63.217	7	24.441	6.610	1	0.600
inst14	104.346	15	21.859	4.976	1	0.464
inst15	99.448	14	20.289	5.223	1	0.424
inst16	96.341	8	30.750	9.482	1	0.580
inst17	71.777	8	19.323	6.349	1	0.630
inst18	65.616	10	31.592	4.953	1	0.375
inst19	66.401	11	30.939	5.582	1	0.485
inst20	146.823	12	32.225	6.594	1	0.518
inst21	201.771	19	29.841	5.839	1	0.520

**Table 15:** Solution quality of the problem instances and pupil assignment strategy 2 for the DARP solver and the OVRP solver.

	DARP				OVRP			
Instance	Quality	Buses	Time loss (max)	Time loss (avg)	Quality	Buses	Time loss (max)	Time loss (avg)
inst01	21.920	3	7.879	3.141	22.860	3	7.879	3.244
inst02	21.661	1	14.304	6.800	21.016	3	11.155	4.528
inst03	16.661	3	9.870	3.454	13.919	3	9.871	2.323
inst04	46.479	2	45.123	13.499	66.504	8	28.536	7.931
inst05	41.806	2	39.447	13.829	60.974	6	22.512	6.833
inst06	42.203	2	38.976	12.724	69.433	8	25.579	5.050
inst07	45.643	5	40.652	13.818	72.915	9	24.861	4.693
inst08	48.192	2	42.239	10.990	86.972	10	20.305	4.108
inst09	50.162	2	36.694	11.194	84.479	10	20.008	3.951
inst10	46.939	1	39.919	11.456	107.341	9	35.113	8.123
inst11	49.938	4	35.394	9.113	127.255	9	34.104	9.175
inst12	61.642	2	38.362	13.430	137.969	13	32.776	6.382
inst13	60.494	3	33.503	12.034	136.504	10	27.474	7.689
inst14	76.739	5	43.523	10.967	157.346	17	23.228	4.077
inst15	77.095	5	38.609	11.112	160.910	15	24.651	5.130
inst16	66.457	3	41.147	11.775	180.139	16	25.919	6.715
inst17	66.583	2	40.479	10.159	198.565	15	27.211	6.307
inst18	67.150	3	39.071	12.819	127.540	12	22.423	6.986
inst19	55.240	3	37.479	12.676	131.504	11	32.794	6.877
inst20	72.619	3	38.362	10.276	280.443	20	31.401	7.522
inst21	74.347	2	43.423	10.705	285.443	21	33.902	6.971

**Table 16:** Solution quality of different problem instances and pupil assignment strategy 3 and unlimited pupil transfers.

			Time loss	Time loss	Transfers	Transfers
Instance	Quality	Buses	(max)	(avg)	(max)	(avg)
inst01	21.824	4	10.288	4.041	2	0.750
inst02	21.016	3	11.155	4.528	0	0.000
inst03	13.919	3	9.871	1.742	0	0.000
inst04	40.643	6	26.146	6.119	1	0.550
inst05	36.776	6	19.091	5.359	1	0.410
inst06	54.767	9	21.952	5.814	2	0.510
inst07	51.929	8	18.553	4.801	1	0.505
inst08	80.629	12	19.384	4.554	1	0.276
inst09	78.908	11	19.378	4.739	2	0.384
inst10	46.666	7	21.180	5.275	2	0.630
inst11	54.845	9	25.215	6.533	2	0.760
inst12	57.245	6	29.441	5.535	2	0.385
inst13	59.472	8	21.583	5.814	2	0.720
inst14	99.886	12	28.070	5.492	3	0.582
inst15	94.493	9	24.472	6.124	2	0.590
inst16	55.662	6	22.339	7.881	2	1.030
inst17	63.715	6	26.274	8.257	4	1.160
inst18	58.357	9	20.310	5.065	2	0.695
inst19	59.044	10	24.843	5.974	3	0.810
inst20	108.432	10	30.047	7.673	3	0.724
inst21	110.443	11	34.495	6.703	3	0.934

**Table 17:** Solution quality of different problem instances and pupil assignment strategy 3 and at most two pupil transfers.

			Time loss	Time loss	Transfers	Transfers
Instance	Quality	Buses	(max)	(avg)	(max)	(avg)
inst01	21.824	3	12.446	5.120	2	0.500
inst02	21.016	3	11.155	4.528	0	0.000
inst03	13.919	3	9.871	1.742	0	0.000
inst04	40.957	7	24.767	5.957	2	0.650
inst05	36.776	6	19.091	5.359	1	0.410
inst06	53.208	12	18.369	4.826	1	0.585
inst07	52.046	10	25.070	5.637	1	0.355
inst08	82.785	12	21.355	4.659	1	0.306
inst09	77.782	10	26.213	4.576	2	0.326
inst10	49.510	5	20.631	6.698	2	0.760
inst11	55.088	11	24.207	7.150	2	0.840
inst12	58.720	8	31.592	6.358	2	0.650
inst13	58.611	9	22.623	5.653	2	0.890
inst14	97.323	13	25.504	4.677	2	0.598
inst15	97.285	11	25.249	5.862	2	0.542
inst16	59.423	8	25.323	8.661	2	1.050
inst17	66.557	6	28.796	7.660	2	0.630
inst18	57.099	8	22.702	5.043	2	0.690
inst19	60.790	10	20.889	4.518	2	0.660
inst20	112.690	10	37.236	7.503	2	0.754
inst21	117.017	9	34.227	7.374	2	0.766

**Table 18:** Solution quality of different problem instances and pupil assignment strategy 3 and at most one pupil transfer.

			Time loss	Time loss	Transfers	Transfers
Instance	Quality	Buses	(max)	(avg)	(max)	(avg)
inst01	22.806	4	5.379	1.861	1	0.250
inst02	21.016	3	11.155	4.528	0	0.000
inst03	13.919	3	9.871	1.742	0	0.000
inst04	40.643	7	25.670	5.739	1	0.470
inst05	37.758	7	19.091	4.633	1	0.310
inst06	53.208	12	18.369	4.826	1	0.590
inst07	52.623	8	18.189	5.007	1	0.470
inst08	80.617	12	20.309	4.524	1	0.300
inst09	80.277	10	17.676	4.125	1	0.300
inst10	51.611	7	20.964	5.030	1	0.520
inst11	60.023	7	22.354	5.464	1	0.500
inst12	61.209	10	19.199	4.808	1	0.560
inst13	62.235	7	24.441	6.745	1	0.590
inst14	103.885	15	23.673	4.493	1	0.420
inst15	98.626	12	23.719	5.521	1	0.440
inst16	102.416	7	27.817	7.560	1	0.550
inst17	86.512	7	32.195	6.143	1	0.400
inst18	58.443	5	38.265	7.724	1	0.470
inst19	64.745	10	30.939	5.823	1	0.480
inst20	133.285	14	32.225	7.592	1	0.550
inst21	134.807	14	29.261	8.443	1	0.580

**Table 19:** Solution quality of the problem instances and pupil assignment strategy 3 for the DARP solver and the OVRP solver.

	DARP				OVRP			
Instance	Quality	Buses	Time loss (max)	Time loss (avg)	Quality	Buses	Time loss (max)	Time loss (avg)
inst01	22.806	3	7.879	2.486	22.806	3	7.879	2.486
inst02	17.956	1	25.627	7.887	21.016	3	11.155	4.528
inst03	13.919	3	9.870	2.523	13.919	3	9.871	2.323
inst04	40.347	1	39.665	12.651	66.504	8	28.536	7.931
inst05	45.793	3	37.113	10.340	60.974	6	22.512	6.833
inst06	45.427	2	39.054	15.995	69.433	8	25.579	5.050
inst07	49.322	2	44.844	14.389	72.915	9	24.861	4.693
inst08	49.696	3	35.602	9.561	86.972	10	20.305	4.108
inst09	48.355	2	41.825	13.918	84.479	10	20.008	3.951
inst10	50.079	2	35.213	13.074	107.341	9	35.113	8.123
inst11	60.882	2	39.695	13.074	127.255	9	34.104	9.175
inst12	69.083	4	37.531	11.605	137.969	13	32.776	6.382
inst13	64.347	2	42.965	13.928	136.504	10	27.474	7.689
inst14	77.824	5	34.748	10.572	157.346	17	23.228	4.077
inst15	70.469	7	42.185	12.904	160.910	15	24.651	5.130
inst16	73.907	5	38.227	12.652	180.139	16	25.919	6.715
inst17	67.210	5	38.117	12.585	198.565	15	27.211	6.307
inst18	65.132	4	37.327	12.651	127.540	12	22.423	6.986
inst19	70.890	4	37.168	11.591	131.504	11	32.794	6.877
inst20	106.800	5	39.071	11.025	280.443	20	31.401	7.522
inst21	82.691	2	42.229	12.008	285.443	21	33.902	6.971

## 7 CONCLUSION AND FURTHER RESEARCH

In this article, a mathematical model, a heuristic solution concept and an in depth analysis of the resulting solution properties for the school bus routing, and scheduling problem with transfers was given. The proposed solution concept has a modular design and can, therefore, be adapted easily in case the problem changes (e.g., if the constraints for the scheduling need to be changed, a general constraint programming solver can replace the current module).

Our computational study investigates three questions. First, it compares solutions of three different modeling approaches. Two approaches without transfers (OVRP, DARP) and our approach (SBR) which allows transfers. The benefit of integrated planning can be seen by comparing the OVRP solutions to solutions calculated by the DARP or SBR solver. For unlimited number of transfers the trade‐off between solution quality, number of transfers, and time loss becomes evident. A high solution quality implies an increase in the time loss if no transfers are allowed. Contrary, if transfers are allowed it is possible to achieve high solution quality with low time loss at the cost of higher average number of transfers. The second analysis shows, that with an increasing number of allowed transfers costs decrease but, consequently, the service level decreases, too. Especially the average number of transfers increases. Third, the computational study investigates the question of how the assignment of pupils can influence solution quality and service level. The impact of the assignment strategy decreases as the number of maximum allowed transfers increases. Again, this comes at the cost of a higher number of transfers. However, in a real‐world scenario, it may not be possible to allow an arbitrary number of pupil transfers.

The proposed solution concept is a first step toward a full decision support system. To further analyze the trade‐off between costs (bus travel time/distance, number of required buses), service level (number of transfers, time loss, walking distance, waiting time), and the influence of problem instance properties a multiobjective optimization approach is needed.
